# Identifying ways to improve diabetes management during cancer treatments (INDICATE): protocol for a qualitative interview study with patients and clinicians

**DOI:** 10.1136/bmjopen-2021-060402

**Published:** 2022-02-22

**Authors:** Laura Ashley, Saifuddin Kassim, Ian Kellar, Lisa Kidd, Frances Mair, Mike Matthews, Mollie Price, Daniel Swinson, Johanna Taylor, Galina Velikova, Jonathan Wadsley

**Affiliations:** 1 Leeds School of Social Sciences, Leeds Beckett University, Leeds, UK; 2 Leeds Centre for Diabetes and Endocrinology, Leeds Teaching Hospitals NHS Trust, Leeds, UK; 3 School of Psychology, University of Leeds, Leeds, UK; 4 Nursing & Healthcare, School of Medicine, Dentistry and Nursing, University of Glasgow, Glasgow, UK; 5 General Practice and Primary Care, Institute of Health and Wellbeing, University of Glasgow, Glasgow, UK; 6 Patient and Public Involvement representative, Harrogate, UK; 7 Leeds Cancer Centre, Leeds Teaching Hospitals NHS Trust, Leeds, UK; 8 Department of Health Sciences, University of York, York, UK; 9 Leeds Institute of Medical Research at St James’s, University of Leeds, Leeds, UK; 10 Weston Park Cancer Centre, Sheffield Teaching Hospitals NHS Foundation Trust, Sheffield, UK

**Keywords:** adult oncology, general diabetes, qualitative research

## Abstract

**Introduction:**

A large and growing number of patients with cancer have comorbid diabetes. Cancer and its treatment can adversely impact glycaemic management and control, and there is accumulating evidence that suboptimal glycaemic control during cancer treatment is a contributory driver of worse cancer-related outcomes in patients with comorbid diabetes. Little research has sought to understand, from the perspective of patients and clinicians, how and why different aspects of cancer care and diabetes care can complicate or facilitate each other, which is key to informing interventions to improve diabetes management during cancer treatments. This study aims to identify and elucidate barriers and enablers to effective diabetes management and control during cancer treatments, and potential intervention targets and strategies to address and harness these, respectively.

**Methods and analysis:**

Qualitative interviews will be conducted with people with diabetes and comorbid cancer (n=30–40) and a range of clinicians (n=30–40) involved in caring for this patient group (eg, oncologists, diabetologists, specialist nurses, general practitioners). Semistructured interviews will examine participants’ experiences of and perspectives on diabetes management and control during cancer treatments. Data will be analysed using framework analysis. Data collection and analysis will be informed by the Theoretical Domains Framework, and related Theory and Techniques Tool and Behaviour Change Wheel, to facilitate examination of a comprehensive range of barriers and enablers and support identification of pertinent and feasible intervention approaches. Study dates: January 2021–January 2023.

**Ethics and dissemination:**

The study has approval from National Health Service (NHS) West Midlands—Edgbaston Research Ethics Committee. Findings will be presented to lay, clinical, academic and NHS and charity service–provider audiences via dissemination of written summaries and presentations, and published in peer-reviewed journals. Findings will be used to inform development and implementation of clinical, health services and patient-management intervention strategies to optimise diabetes management and control during cancer treatments.

**Strengths and limitations of this study**

- Largest, and first UK-based, qualitative study to date of patients’ and clinicians’ views on barriers and enablers to effective diabetes management during cancer treatments.
- Most in-depth qualitative examination of this topic to date, with a focus on both barriers and enablers, and ways to address and harness these, respectively, for both patient and clinician diabetes management during cancer treatments.
- Will extend previous research by interviewing a wider range of clinicians (crucially including diabetologists) and considering other cancer treatments in addition to chemotherapy.
- Data collection and analysis will be informed by the Theoretical Domains Framework, and related Theory and Techniques Tool and Behaviour Change Wheel, to facilitate examination of a comprehensive range of barriers and enablers and support identification of feasible intervention approaches.
- Recruitment is limited, in the main, to just two sites, both Yorkshire based.

## Introduction

### A large and growing number of patients with cancer have comorbid diabetes

The incidence and prevalence of diabetes mellitus are high and increasing worldwide.^1 2^ In high-income countries, it is estimated 87%–91% of all people with diabetes have type 2 diabetes, 7%–12% type 1 and 1%–3% other rarer types of diabetes.^1^ In the UK, it is estimated that 4.8 million people are living with diabetes, projected to increase to 5.3 million by 2025.^3^ Type 1 and type 2 diabetes are associated with an increased risk of cancer, including, collectively, cancers of the breast, colorectum, endometrium and oesophagus.^4–7^ In the UK, one in two people now develop cancer in their lifetime.^8^ Figures on the prevalence of pre-existing diabetes in newly diagnosed patients with cancer vary (eg, by country, cancer type) but studies typically report rates ranging from 10% to 20%.^9–12^.

### Cancer and its treatment can adversely impact glycaemic management and control

For people with diabetes, maintaining good glycaemic control can be a significant challenge.^13 14^ This challenge may be exacerbated by cancer and its treatment, which has high potential to complicate diabetes management and glycaemic control. The psychosocial sequelae of a cancer diagnosis (eg, distress, anxiety, depression^15–17^) and the side-effects of some cancer treatments (eg, vomiting, fatigue, pain,^18 19^) could impede diabetes management behaviours such as healthy eating and blood glucose monitoring. Furthermore, some cancer and supportive treatments can directly impact blood sugar levels increasing the risk of hypoglycaemic and hyperglycaemic episodes (eg, somatostatin analogues, high-dose steroids). Though results are mixed, studies collectively indicate a deleterious effect of cancer and its treatment on diabetes management and control, which for many people is suboptimal even before diagnosis of cancer.^13 14^ Research shows that during cancer treatment many people with diabetes have reduced adherence to diabetes medications and self-care behaviours that contribute to glycaemic control (eg, blood glucose monitoring, eating healthily, exercising), and have poorer glycaemic control (eg, increased HbA1c levels, diabetes treatment escalations), than pre-cancer diagnosis.^20 21^ Studies have also noted that, following a cancer diagnosis, some people with diabetes undergo less diabetes-related screening aimed at mitigation of diabetic complications (eg, retinal screening, low-density lipoprotein tests).^22–24^

### Suboptimal diabetes management and control during cancer treatment is associated with worse outcomes

Some studies have found patients with cancer with pre-existing diabetes are more likely to experience toxicities and complications (eg, infections) during cancer treatment,^25–31^ which can result in costly hospitalisations and compromise treatment completion. Moreover, numerous studies show that, compared with other patients with cancer, those with pre-existing diabetes have higher perioperative and longer term mortality.^6 25–27 30–34^ Though findings are not uniform, there is accumulating evidence that suboptimal glycaemic control during cancer treatment is a contributory driver of worse cancer-related outcomes in patients with comorbid diabetes.^28 35–41^ Retrospective studies of patients with cancer with comorbid diabetes have shown, for example, that good perioperative glycaemic control is associated with reduced risk of morbidity and death following colectomy for colon cancer^35^ and good glycaemic control prior to neoadjuvant chemotherapy for cervical cancer is associated with superior tumour response and survival.^37^ A small 12-week prospective study of patients with cancer with pre-existing diabetes found suboptimal glycaemic control at chemotherapy outset predicted increased risk of developing an infection, hospitalisation and chemotherapy reduction or discontinuation.^41^ Additionally, suboptimal glycaemic control can cause unpleasant symptoms (eg, fatigue, inability to concentrate, increased thirst) which may reduce health-related quality of life (HRQoL). Indeed, a recent study found patients with cancer with diabetes who had suboptimal glycaemic control reported poorer HRQoL during chemotherapy than both patients with cancer without diabetes and those with diabetes who had good glycaemic control.^42^ Also, declines in glycaemic control and diabetes-related screening during the period of a cancer diagnosis and treatment could increase the risk of diabetic complications such as retinopathy and cardiovascular events. A study in Canada found that, compared with patients with diabetes without cancer, patients with diabetes with cancer had significantly more hospital visits for diabetic emergencies, skin and soft-tissue infections, and cardiovascular events in the year following their cancer diagnosis.^43^

### Few studies have examined patients’ and clinicians’ views on barriers and enablers to effective diabetes management during cancer treatments

Little research has sought to identify and understand the barriers and enablers to effective diabetes management and control during cancer treatments. Understanding how and why different aspects of cancer care and diabetes care can complicate or facilitate each other, from the perspective of patients and clinicians, is key to informing clinical, health services and patient-management interventions to improve diabetes management during cancer treatments. In a survey of people with diabetes (n=37), Hershey *et al*^44 45^ found that patient-reported reductions in diabetes self-management activities during cancer chemotherapy were associated with greater symptom burden and lower diabetes self-efficacy. Hershey *et al*’s survey also included two open-ended questions about the impact of cancer on diabetes, which revealed that many patients prioritised cancer care over diabetes care, with some reporting advice from primary-care providers not to be concerned with diabetes during chemotherapy. In a focus-group with patients (n=5), Hershey *et al*^46^ similarly found that patients reported that cancer treatment took priority. Hershey *et al* also conducted focus groups with oncology clinicians (n=20),^46^ finding that oncologists generally saw diabetes management to be outside their remit, and the responsibility of primary care, but noted poor communication between oncology and primary care. These findings were recently corroborated by Cho *et al*,^47^ who interviewed oncologists (n=10) and primary care doctors and nurses (n=10) about diabetes management during cancer treatment. Cho *et al* found both oncology and primary care providers thought primary care should be responsible for diabetes care, though noted barriers to this including very infrequent and limited communication between oncology and primary care, and the fact that many patients reduce contact with primary care following a cancer diagnosis. Though these qualitative studies provide important insights into the challenges of managing diabetes during cancer treatments, they have involved a relatively small number of patients and clinicians, and are limited in scope and depth. Hershey’s studies with patients^44–46^ both focused on only chemotherapy cancer treatment, and predominately surveyed participants during this treatment, meaning they could not obtain perspectives on other elements of cancer treatment (eg, radiotherapy, long-term tamoxifen) or with the benefit of reflection (ie, looking back on completed treatment). Furthermore, with just two open-ended questions, Hershey *et al*’s survey study did not undertake an in-depth examination of participants’ experiences. The studies with clinicians by Hershey Hershey *et al*^46^ and Cho *et al*^47^ both focused exclusively on type 2 diabetes and do not include the perspectives of several professions relevant to diabetes management during cancer, including diabetes doctors and specialist nurses, anaesthetists and dieticians. Moreover, these prior studies have focused on barriers to diabetes management during cancer treatments, with limited or no focus on patients’ and clinicians’ perspectives on enabling factors and potential interventions. Also, current studies are exclusively USA based, and findings may to some degree be context specific, given differences in the organisation and financing of heathcare systems globally, even within higher income countries. We aim to extend, and address some of the limitations of, this previous qualitative work.

### Our study aims to extend previous qualitative work in this area and help inform intervention development

The current qualitative interview study aims to identify and elucidate challenges and enablers to diabetes management and control during treatment for cancer, based on the experiences and perspectives of people with diabetes and comorbid cancer and healthcare professionals involved in their care. To facilitate examination of a comprehensive range of individual-level and service-level barriers and enablers, and to support the identification of potential pertinent intervention approaches to address and harness these, respectively, we will use the Theoretical Domains Framework (TDF),^48 49^ and related Theory and Techniques Tool (TTT)^50–53^ and Behaviour Change Wheel (BCW),^54 55^ to inform data collection and analysis, as detailed in the Methods section.

### Research questions

1. What are the patient-perceived challenges and enablers to effective self-management and control of diabetes during cancer treatments?
2. What are the clinician-perceived challenges and enablers to effective clinical management and control of diabetes during cancer treatments?
3. What are patients’ suggestions for ways to support and improve self-management and control of diabetes during cancer treatments?
4. What are clinicians’ suggestions for ways to support and improve clinical management and control of diabetes during cancer treatments?
5. What are potentially promising intervention targets and strategies for consideration in future research to optimise patient and/or clinician management and control of diabetes during cancer treatments?

## Methods and analysis

### Participants

Eligibility criteria are detailed in table 1. We will include patients with type 1 or type 2 diabetes, and clinician interviews will enquire about differences between these patient groups. This will enable us to examine in the analysis similarities and differences in patient and clinician reported challenges and enablers to diabetes management during cancer treatments on the basis of diabetes type, and thus help inform to what extent different future interventions in this area could address both diabetes types or may need to target one or both of the types separately. We will restrict recruitment to comorbid breast, prostate or colorectal cancer; in the UK, these are three of the four most common cancers and the largest survivor groups.^56^

**Table 1:** Participant eligibility criteria

	Inclusion	Exclusion
Patients	Medically diagnosed type 1 or type 2 diabetes Subsequent diagnosis of breast, prostate or colorectal cancer Received any type of localised or systemic National Health Service anti-cancer treatment (currently or within the last 3 years)	Under 18 years of age Clinician-estimated life expectancy of less than 3 months Lack capacity to provide informed consent
Clinicians	Involved in providing care to above comorbid patient group (ie, patients with cancer with pre-existing diabetes) in relation to their diabetes and/or cancer	

### Recruitment sites and procedures

#### Patients

##### Hospital-based recruitment

Patients will be primarily recruited from cancer centres in two Yorkshire-based National Health Service (NHS) Hospital Trusts. Clinical teams, possibly with Clinical Research Network support, will identify eligible patients and first approach them about the study, providing a patient information sheet. Interested patients will contact our research team directly, or, if a patient requests it, the clinical team will pass onto us patient-provided contact information and we will initiate correspondence.

##### Community-advertisement recruitment

We will also recruit patients via an advertisement flyer calling for people aged 18+ ‘with diabetes (type 1 or type 2), who are being treated for breast, bowel or prostate cancer, or have been in the past 3 years’ to ‘tell us about your experiences of managing diabetes during cancer treatments’. The flyer will be disseminated via social media (eg, Twitter and Facebook accounts of the research team members), and by relevant willing UK-based charities and organisations (eg, in newsletters). Patients who see the flyer and are interested will contact our research team directly.

#### Clinicians

##### Hospital-based recruitment

Clinicians will be recruited from the cancer centres, as well as other relevant hospital departments and specialties (eg, endocrinology, anaesthesia, pharmacy, dietetics) within the participating hospitals. Our research team includes oncology and diabetes clinicians working at the participating hospital trusts. Potentially eligible clinicians will be identified and emailed a staff information sheet by a member of the research team, or by a gatekeeper colleague who has agreed to disseminate study information. Eligible and interested clinicians will contact the research team directly.

##### Primary care recruitment

We will also recruit general practitioners (GPs) and practice nurses working at general practices in Yorkshire. GPs and practice nurses will be informed about the study by local primary care R&D teams who are willing to disseminate study information (eg, in a CCG-newsletter), or will be emailed a staff information sheet by a member of the research team (eg, GPs with a part-time academic post known to the research team). Eligible and interested clinicians will contact the research team directly.

### Sample size and sampling strategy

We will recruit and interview 30–40 patients and 30–40 clinicians. Based on our experience, this sample size will enable adequate sample diversity on key participant characteristics and allow us to reach sufficient data saturation. We aim to recruit comparable numbers of people with breast, prostate and colorectal cancer (n≈10–13 each cancer type), with both diabetes types represented in each cancer subgroup (2–3 patients in each cancer subgroup with type 1 diabetes, which accounts for 7%–12% of all diabetes cases in high-income countries^1^). We also aim for some diversity in the sample as a whole with regard to types of cancer treatment, sociodemographic characteristics (gender, age, ethnicity), and the extent of experienced difficulties with diabetes management during cancer treatments. We aim to recruit clinicians from a wide range of relevant professions and specialities (eg, oncologists, surgeons, diabetologists, specialist nurses, dieticians, GPs) and for some diversity in the sample as a whole with regard to professional seniority. The composition of the sample will be monitored during recruitment and, if necessary and possible, targeted recruitment of under-represented groups will be undertaken.

### Theoretical framework informing data collection and analysis

We will use the TDF^48 49^ and related resources to inform data collection and analysis. The TDF synthesises key constructs in numerous theories of behaviour and behaviour change, and thus ‘provides a theoretical lens through which to view the cognitive, affective, social and environmental influences on behaviour’ (p2).^57^ The TDF version-2 comprises 84 constructs theorised to influence behaviour (eg, professional identity; self-efficacy; cognitive overload/tiredness) organised by 14 domains (eg, social/professional role and identity; beliefs about consequences; environmental context and resources). These domains can be considered Mechanisms of Action (MoA), that is processes which influence behaviour, and are thus potential intervention targets. The TDF is part of an evolving set of resources being developed by Michie *et al* to promote design of more effective theory-based behavioural interventions. These resources include the TTT,^50–53^ which provides guidance on linking MoAs to pertinent Behaviour Change Techniques (BCTs) (eg, verbal persuasion about capability; conserving mental resources; information about health consequences), which are the potentially ‘active ingredients’ in an intervention that changes behaviour. The TTT synthesises the evidence (or not) for links between 74 BCTs and 26 MoAs, which include the 14 TDF domains and the 12 most frequently occurring MoAs which did not overlap with these identified in a review of behaviour change theories.^52^ The BCW^54 55^ is a ‘theory and evidence based’ intervention development approach^58^ that provides guidance on considering and identifying the function of interventions (eg, education, persuasion, enablement) and policy categories that may support the delivery of these functions (eg, guidelines, service provision, environmental planning) and how these both link to MoAs and/or BCTs. The TDF and related resources have been used to inform the development and evaluation of health-focused behavioural interventions,^59–61^ including informing the data collection and/or analysis of qualitative studies forming an early stage in the process of intervention development.^62–65^

### Data collection: interview content and procedures

Participants will take part in one semistructured qualitative interview lasting around 45 min, though duration is likely to be variable depending on how much a participant wishes to say and their preferred interview pace. Participants can choose their interview date/time and mode (eg, telephone, videocall, in person) provided arrangements adhere to current relevant government and workplace rules around COVID-19 social distancing. Interviews will examine participants’ experiences of and perspectives on diabetes management and control during cancer treatments. Early interview questions will enquire about key sociodemographic characteristics and relevant clinical (eg, diabetes and cancer type and treatments) or professional (eg, job-title, workplace) details. Interviews will seek to identify and elucidate patient-perceived and clinician-perceived challenges and enablers to effective diabetes management and glycaemic control in the context of cancer diagnosis and treatment, and ways to address and optimise these, respectively. Interview guides were developed, informed by: (1) previous research^44–47^; (2) advice and feedback from the study patient and public involvement (PPI) and steering groups; and lastly (3) the MoA covered by the TTT^50^ which, as previously discussed, includes the TDF domains. We were mindful interviews do not become dominated by examining MoAs, especially given multiple behaviours are involved in managing diabetes (eg, prescribing medications, following dietary advice, blood glucose self-monitoring) and to systematically examine each MoA in relation to each different behaviour that may be discussed would make for an overly long, repetitive, and granular-level interview. Also, we did not want interviews to be restricted to examining only the MoAs, thereby potentially overlooking other influential factors. Thus, in an approach consistent with recent recommendations for using the TDF in qualitative studies,^66^ the interview guides contained at least one question or follow-up question likely to encourage discussion of challenges or enablers relevant to each MoA, rather than a highly structured list of MoA-focused questions, one per MoA per different behaviours. Pilot interviews were undertaken with three clinician coapplicants and three PPI group members, to refine the interview guides and hone interviewer technique. Box 1 shows sample questions from the patient and clinician interview guides. Interviews will be audio recorded.

Box 1 **Sample interview questions from the interview guides**

**Patient interviews**

*Selected opening questions to key topic areas and example follow-up questions*

- In what ways, if any, did your diabetes management change during the time that you were/are having cancer treatment?
  - any changes to the foods you ate?
- In terms of managing your diabetes during cancer treatments, what have you found to be your biggest challenges?
  - has it been difficult to remember (eg, to take tablets, self-monitor blood glucose) during treatment?
- How important do you feel it is to effectively manage your diabetes throughout cancer treatments?
  - is it more or less important to you than before cancer?
- How possible do you think it is to manage diabetes well while having cancer treatments?
  - because of the work/time/energy involved in undergoing cancer treatments?
- Did you receive any information about diabetes management during cancer treatments?
  - from who, when?
- What has the support been like from healthcare professionals in terms of managing your diabetes during the time that you were having cancer treatment?
  - how important did it seem to your (eg, oncologist, general practitioner (GP)) to manage your diabetes well during this time?
- Have any family or friends been involved in helping you to manage your diabetes during the time you were having cancer treatment?
  - how do they help?
- What do you think could be done to help people to manage their diabetes better during the time they are having cancer treatment?
  - why would that help?

*Example cross-topic follow-up questions*

- is that something you know about?
- is that something you know how to do?
- is that something that would be easy or difficult to do?
- what would prompt you to do that?
- what would be the reasons to do/not do that?
- and do you think your family and friends/doctors tend to think similarly?
- what would help you with that?

**Clinician interviews**

*Selected opening questions to key topic areas and example follow-up questions*

- Research suggests that managing diabetes can be difficult during cancer treatments, is this something you see/encounter in your role?
  - are there differences in the difficulties between patients with type 1 and type 2 diabetes?
- As a (eg, medical oncologist, GP), what do you see as your role in supporting clinical management of diabetes during cancer treatments?
  - and in supporting patients’ self-management of their diabetes?
- Can you tell me about how you identify this patient group—that you know you are dealing with a patient who has both cancer *and* diabetes
  - once you know a patient has diabetes/is having cancer treatment, would it cause you to do anything differently?
- How important do you feel it is to effectively manage diabetes during a time that someone is also having cancer treatments?
  - what do you see are the benefits of good/risks of poor diabetes management during cancer treatments?
- Are there any care protocols or clinical guidelines you follow when caring and making decisions for this group?
  - do you find these useful?
- Do patients receive any information about or support with diabetes management during cancer treatments?
  - do patients tend to ask for information/help?
- If there are difficulties controlling a patients’ diabetes during cancer treatment what sorts of things would be done to address this?
  - what about altering medications/bringing other professionals in/trying to improve patient self-management?
- Thinking of your work environment, what improvements would make it easier for you to support good diabetes management for this group?
  - what about resources/recording and reporting systems/workflows and processes/culture?

*Example cross-topic follow-up questions*

- is that something you know how to do?
- is that something that would be easy or difficult to do?
- is that something you feel confident to do?
- is that something you always/usually/often do?
- how would you know if that had been done?
- and do you think your colleagues tend to think/do similarly?
- what would need to happen for you to do that/be able to do that?

### Data analysis

#### Primary

The interview data will be analysed to identify and elucidate patient and clinician perceived challenges and enablers to self and clinical diabetes management and control during cancer treatments, and suggested ways to overcome and optimise these respectively. We will use the framework method,^67^ guided by the stages and recommendations set out by Gale *et al*^68^: (1) verbatim transcription; (2) familiarisation with the data; (3) coding; (4) developing a working analytical framework; (5) applying the framework; (6) charting data into a framework matrix (a summary of the data by analytic code/category per participant); and (7) interpreting the data. Analysis will overlap with data collection. To ensure rigour, analysis will be an iterative, collaborative process led by the core research team members (LA, IK, LK, MP, JT), who have experience of framework analysis and using the TDF in qualitative research,^63 69 70^ with input and feedback from other members of the research team and the PPI and steering groups at key points. We will use QSR-NVivo software to support analysis, and document substantive decisions during the analytic process. Coding will use both deductive codes (ie, based on the research questions, interview guides) and inductive codes (ie, based on reading a sample of transcripts), which will be generated through iterative rounds of independent work and subsequent group discussion. In line with recent recommendations for using the TDF in qualitative studies,^66^ we do not intend to include the MoAs as a priori codes, but to subsequently consider inductively generated findings about challenges and enablers against the MoAs (see below). This will guard against overlooking challenges and enablers discussed in the interviews not covered by the TTT and, as the TTT includes a substantial number of MoAs (n=26), guard against other key focuses of the analysis being overshadowed (eg, interviewees suggestions for tackling challenges). A working analytical framework will be iteratively developed through agreeing by consensus on a final set of codes, and their initial organisation into categories, informed by input from the PPI group and wider research team. Members of the PPI group will each read a different sample of transcripts and provide coding-relevant feedback (eg, feedback on sections that stood out to them, on barriers and enablers discussed in the interview). Other members of the research team and steering group will be asked to review and feedback on a draft(s) of the working analytical framework. The analytical framework will be applied to all interview transcripts, and subsequently the data will be charted into a framework matrix, including references to illustrative quotations. A proportion of transcripts will be double coded and double charted (≈10% at both stages), and compared and discussed, to ensure consistent application of the framework and data charting. We will identify and develop themes and subthemes pertinent to the research questions, using the matrix to facilitate comparison within and between codes/categories and participants, and thus the identification of patterns and deviant cases in the data, including on the basis of diabetes and/or cancer type and/or treatments. Members of the PPI group, wider research team and steering group will input into data interpretation by reviewing and providing feedback on the matrix and a related working draft(s) of themes and subthemes. We envisage the patient and clinician data will initially be coded and charted separately, but subsequently synthesised as much as possible during interpretation and theme development. However, decisions about this aspect of the analysis will be made during analysis, after familiarisation with the data.

#### Secondary

In a second phase of analysis we will: (1) map the challenges and enablers identified in the framework analysis phase to the MoAs; (2) prioritise MoAs for targeting in interventions; and (3) determine pertinent intervention functions and BCTs to deliver these. We will map to the MoAs using current definitions,^50^ and by independent work followed by group discussion to achieve a consensus. Target MoAs will be prioritised through consensus discussion, on the basis of the mapping results, the contextualised understanding of barriers and ways to address these provided by the framework analysis of the interview data, and views of the study PPI group and wider research team and steering group (which include multidisciplinary clinicians). For each prioritised MoA, we will determine, through consensus discussion, potential intervention functions and policy categories using the BCW^54 55^ and pertinent BCTs using the TTT.^50^ These processes are consistent with those undertaken in previous qualitative studies, including by members of our team, which have similarly sought to understand, and inform interventions to address, healthcare challenges (eg, gestational diabetes^62^; deprescribing^63^; smoking in pregnancy^65^).

### Patient and public involvement

Six PPI representatives, with personal experience of diabetes, cancer or both conditions as a patient or carer, were involved in developing this research and helped shape the design and methods of the study. One of these PPI representatives was a coapplicant on our grant application for funding and is a coauthor of this protocol paper. We have established a study PPI group with six members, one of whom, coapplicant and protocol coauthor MM, was involved in the previous study development stage. As well as PPI-group meetings, MM will also attend study steering group meetings, helping ensure effective communication between these two groups. The PPI group will collaborate, advise and feedback on all stages of the research including: design and piloting of the interview guides; data analysis including coding and interpreting the data; and dissemination outputs including lay summaries, presentations and journal papers. We will discuss with PPI group members the research activities they wish to be involved in, their relevant prior experience (if any), and therefore what training they may require (eg, practising data coding using transcripts from the pilot interviews), and arrange as and when appropriate. We will be guided by the UK Standards for Public Involvement,^71^ and PPI representatives will be paid following UK National Institute for Health Research guidance^72^ and reimbursed any expenses.

## Ethics and dissemination

### Approvals and ethical considerations

The study has approval from the NHS West Midlands—Edgbaston Research Ethics Committee (20/WM/0310), NHS Health Research Authority (IRAS-ID:276694) and the Leeds Beckett University Psychology Research Ethics Committee. Participants will receive written study information which will include contact details of organisations providing diabetes-related and cancer-related information and support (eg, Macmillan helpline). Consent will be obtained in writing or verbally, depending on participant preference; verbal consent will be recorded immediately prior to the interview and stored on a separate audio file to the interview. Participants will be informed of their right to choose, without needing to provide a reason why, to not answer an interview question(s), to take a break or stop the interview at any time, and to request withdrawal of any/all of their data. Research and personal data will be handled and stored confidentially and securely in line with government and Leeds Beckett University data protection requirements and guidelines. We will protect participant anonymity and make unidentifiable all illustrative quotes used in reports of the findings. Participants will not be offered any incentives.

### Dissemination

Findings will be presented to lay, clinical, academic and NHS and charity service provider audiences via dissemination of written summaries and articles, infographics and presentations. Findings will be published in a peer-reviewed journal(s) following Consolidated criteria for Reporting Qualitative research and Standards for Reporting Qualitative Research reporting guidelinesonline supplemental file 1 .^73 74^ Study data may be made available on reasonable request to the corresponding author. This UK-based study will be the first published study of its kind outside the USA, and will provide the most in-depth qualitative examination of this topic to date, with a focus on both barriers and enablers, and ways to address and harness these, respectively, for both patient and clinician diabetes management during cancer treatments. Our study will extend previous work by also interviewing a wider range of clinicians, crucially including diabetologists, and considering other cancer treatments in addition to chemotherapy. Furthermore, while previous work in this area has not been theoretically informed, at least explicitly, we will use the TDF, TTT and BCW^48–55^ to facilitate examination of a comprehensive range of barriers and enablers and support identification of pertinent and feasible intervention approaches. There is increasing interest worldwide in improving glycaemic control during cancer treatments, evident in the recent development of interventions for people with diabetes having treatment for cancer, such as clinical guidelines in UK,^75^ new integrated care pathways in Italy,^76^ and a clinical pharmacy intervention and counselling programme in Turkey,^77^ though intervention research in this area is in its infancy. Findings of this study will be used to inform development and implementation of clinical, health services and patient-management intervention strategies to optimise diabetes management and control during cancer treatments.

10.1136/bmjopen-2021-060402.supp1 Supplementary data

### Timeline

Data collection commenced February 2021 and is projected to close August 2022.

## Supplementary Material

Reviewer comments

Author's manuscript
