# Supplementary material for: Identifying ways to improve diabetes management during cancer treatments (INDICATE): protocol for a qualitative interview study with patients and clinicians
Source: BMJ Open. 2022 Feb 22;12(2):e060402. doi: 10.1136/bmjopen-2021-060402 (PMC8867345; doi:10.1136/bmjopen-2021-060402)
Supplement: Author's manuscript [file bmjopen-2021-060402.draft_revisions.pdf]

BMJ Open is committed to open peer review. As part of this commitment we make the peer review history of every article we publish publicly available.

When an article is published we post the peer reviewers' comments and the authors' responses online. We also post the versions of the paper that were used during peer review. These are the versions that the peer review comments apply to.

The versions of the paper that follow are the versions that were submitted during the peer review process. They are not the versions of record or the final published versions. They should not be cited or distributed as the published version of this manuscript.

BMJ Open is an open access journal and the full, final, typeset and author-corrected version of record of the manuscript is available on our site with no access controls, subscription charges or pay-per-view fees (http://bmjopen.bmj.com).

If you have any questions on BMJ Open's open peer review process please email info.bmjopen@bmj.com

BMJ Open

Identifying ways to improve diabetes management during cancer treatments (INDICATE): protocol for a qualitative interview study with patients and clinicians

Journal:	*BMJ Open*
Manuscript ID	bmjopen-2021-060402
Article Type:	Protocol
Date Submitted by the Author:	20-Dec-2021

Complete List of Authors:	Ashley, Laura; Leeds Beckett University,<br>Kassim, Saifuddin ; Leeds Teaching Hospitals NHS Trust, Leeds Centre for Diabetes and Endocrinology<br>Kellar, Ian; Bradford Institute for Health Reserach, Yorkshire Quality and Safety Research Group<br>Kidd, Lisa; University of Glasgow, Nursing & Healthcare, School of Medicine, Dentistry and Nursing; University of Glasgow<br>Mair, Frances; University of Glasgow, General Practice and Primary Care<br>Matthews, Mike; PPI-representative<br>Price, Mollie; Leeds Beckett University<br>Swinson, Daniel; St James's University Hospital, Leeds Cancer Centre;<br>Taylor, Johanna; Mental Health and Addiction Research Group, Department of Health Sciences, University of York<br>Velikova, Galina; University of Leeds, Leeds Institute of Medical Research<br>Wadsley, Jonathan; Weston Park Hospital,

Keywords:	Adult oncology < ONCOLOGY, General diabetes < DIABETES & ENDOCRINOLOGY, QUALITATIVE RESEARCH

SCHOLARONE™
Manuscripts

Identifying ways to improve diabetes management during cancer treatments (INDICATE): protocol for a qualitative interview study with patients and clinicians

Laura Ashley[1], Saifuddin Kassim[2], Ian Kellar[3], Lisa Kidd[4], Frances S Mair[5], Michael Matthews[6], Mollie Price[1], Daniel Swinson[7], Johanna Taylor[8], Galina Velikova[7,9], Jonathan Wadsley[10,11]

[1]Leeds School of Social Sciences, Leeds Beckett University, Leeds, UK

[2]Leeds Centre for Diabetes and Endocrinology, Leeds Teaching Hospitals NHS Trust, Leeds, UK

[3]School of Psychology, University of Leeds, Leeds, UK

[4]School of Medicine, Dentistry & Nursing, University of Glasgow, Glasgow, UK

[5]Institute of Health and Wellbeing, University of Glasgow, Glasgow, UK

[6]Patient and Public Involvement (PPI) representative, Harrogate, UK

[7]Leeds Cancer Centre, Leeds Teaching Hospitals NHS Trust, Leeds, UK

[8]Department of Health Sciences, University of York, York, UK

[9]Leeds Institute of Medical Research at St. James's, School of Medicine, University of Leeds, Leeds, UK

[10]Weston Park Cancer Centre, Sheffield Teaching Hospitals NHS Foundation Trust, Sheffield, UK

[11]Deaprtment of Oncology and Metabolism, University of Sheffield, Sheffield, UK

Correspondence to

Dr Laura Ashley

Rm CL821, Calverley Building, City Campus, Leeds Beckett University, Leeds, LS1 3HE, UK

L.J.Ashley@leedsbeckett.ac.uk

Twitter: @Dr_Laura_Ashley

Word count: 4,000

For peer review only

ABSTRACT

Introduction

A large and growing number of cancer patients have comorbid diabetes. Cancer and its treatment can adversely impact glycemic management and control, and there is accumulating evidence that suboptimal glycemic control during cancer treatment is a contributory driver of worse cancer-related outcomes in patients with comorbid diabetes. Little research has sought to understand, from the perspective of patients and clinicians, how and why different aspects of cancer care and diabetes care can complicate or facilitate each other, which is key to informing interventions to improve diabetes management during cancer treatments. This study aims to identify and elucidate barriers and enablers to effective diabetes management and control during cancer treatments, and potential intervention targets and strategies to address and harness these respectively.

Methods and analysis

Qualitative interviews will be conducted with people with diabetes and comorbid cancer (n=30-40) and a range of clinicians (n=30-40) involved in caring for this patient group (e.g., oncologists, diabetologists, specialist nurses, general practitioners). Semi-structured interviews will examine participants' experiences of and perspectives on diabetes management and control during cancer treatments. Data will be analysed using Framework analysis. Data collection and analysis will be informed by the Theoretical Domains Framework, and related Theory and Techniques Tool and Behaviour Change Wheel, to facilitate examination of a comprehensive range of barriers and enablers and support identification of pertinent and feasible intervention approaches. Study dates: January 2021 – January 2023.

Ethics and dissemination

The study has approval from NHS West Midlands – Edgbaston Research Ethics Committee. Findings will be presented to lay, clinical, academic and NHS and charity service-provider audiences via dissemination of written summaries and presentations, and published in peer-reviewed journals. Findings will be used to inform development and implementation of clinical, health services and patient-management intervention strategies to optimise diabetes management and control during cancer treatments.

For peer review only

Strengths and limitations of this study

- Largest, and first UK-based, qualitative study to date of patients' and clinicians' views on barriers and enablers to effective diabetes management during cancer treatments.
- Most in-depth qualitative examination of this topic to date, with a focus on both barriers and enablers, and ways to address and harness these respectively, for both patient and clinician diabetes management during cancer treatments.
- Will extend previous research by interviewing a wider range of clinicians (crucially including diabetologists) and considering other cancer treatments in addition to chemotherapy.
- Data collection and analysis will be informed by the Theoretical Domains Framework, and related Theory and Techniques Tool and Behaviour Change Wheel, to facilitate examination of a comprehensive range of barriers and enablers and support identification of feasible intervention approaches.
- Recruitment is predominantly via two sites, both Yorkshire-based.

For peer review only

INTRODUCTION

A large and growing number of cancer patients have comorbid diabetes

The incidence and prevalence of diabetes mellitus are high and increasing worldwide [1,2]. In high-income countries, it is estimated 87-91% of all people with diabetes have type 2 diabetes, 7-12% type 1, and 1-3% other rarer types of diabetes [1]. In the United Kingdom (UK), it is estimated that 4.8 million people are living with diabetes, projected to increase to 5.3 million by 2025 [3]. Type 1 and type 2 diabetes are associated with an increased risk of cancer, including, collectively, cancers of the breast, colorectum, endometrium and oesophagus [4-7]. In the UK, one in two people now develop cancer in their lifetime [8]. Figures on the prevalence of pre-existing diabetes in newly diagnosed cancer patients vary (e.g., by country, cancer type) but studies typically report rates ranging from 10-20% [e.g.,9-12].

Cancer and its treatment can adversely impact glycemic management and control

For people with diabetes, maintaining good glycemic control can be a significant challenge [13,14]. This challenge may be exacerbated by cancer and its treatment, which has high potential to complicate diabetes management and glycemic control. The psychosocial sequelae of a cancer diagnosis (e.g., distress, anxiety, depression [15-17]) and the side-effects of some cancer treatments (e.g., vomiting, fatigue, pain, [18,19]) could impede diabetes management behaviours such as healthy eating and blood glucose monitoring. Furthermore, some cancer and supportive treatments can directly impact blood sugar levels increasing the risk of hypo- and hyperglycemic episodes (e.g., somatostatin analogues, high-dose steroids). Though results are mixed, studies collectively indicate a deleterious effect of cancer and its treatment on diabetes management and control, which for many people is suboptimal even before diagnosis of cancer [13,14]. Research shows that during cancer treatment many people with diabetes have reduced adherence to diabetes medications and self-care behaviours that contribute to glycemic control (e.g., blood glucose monitoring, eating healthily, exercising), and have poorer glycemic control (e.g., increased HbA1c levels, diabetes treatment escalations), than pre-cancer diagnosis [20,21]. Studies have also noted that, following a cancer diagnosis, some people with diabetes undergo less diabetes-related screening aimed at mitigation of diabetic complications (e.g., retinal screening, low-density lipoprotein tests) [22-24].

Suboptimal diabetes management and control during cancer treatment is associated with worse outcomes

Some studies have found cancer patients with pre-existing diabetes are more likely to experience toxicities and complications (e.g., infections) during cancer treatment [25-31], which can result in costly hospitalisations and compromise treatment completion. Moreover, numerous studies show that, compared to other cancer patients, those with pre-existing diabetes have higher perioperative and longer-term mortality [6,25-27,30-34]. Though findings are not uniform, there is accumulating evidence that suboptimal glycemic control during cancer treatment is a contributory driver of worse cancer-related outcomes in patients with comorbid diabetes [28,35-41]. Retrospective studies of cancer patients with comorbid diabetes have shown, for example, that good perioperative glycemic control is associated with reduced risk of morbidity and death following colectomy for colon cancer [35] and good glycemic control prior to neoadjuvant chemotherapy for cervical cancer is associated with superior tumour response and survival [37]. A small 12-week prospective study of cancer

For peer review only

patients with pre-existing diabetes found suboptimal glycemic control at chemotherapy outset predicted increased risk of developing an infection, hospitalisation, and chemotherapy reduction or discontinuation [41].

Additionally, suboptimal glycemic control can cause unpleasant symptoms (e.g., fatigue, inability to concentrate, increased thirst) which may reduce health-related quality of life (HRQoL). Indeed, a recent study found cancer patients with diabetes who had suboptimal glycemic control reported poorer HRQoL during chemotherapy than both cancer patients without diabetes and those with diabetes who had good glycemic control [42]. Also, declines in glycemic control and diabetes-related screening during the period of a cancer diagnosis and treatment could increase the risk of diabetic complications such as retinopathy and cardiovascular events. A study in Canada found that, compared to diabetes patients without cancer, diabetes patients with cancer had significantly more hospital visits for diabetic emergencies, skin and soft tissue infections, and cardiovascular events in the year following their cancer diagnosis [43].

Few studies have examined patients' and clinicians' views on barriers and enablers to effective diabetes management during cancer treatments

Little research has sought to identify and understand the barriers and enablers to effective diabetes management and control during cancer treatments. Understanding how and why different aspects of cancer care and diabetes care can complicate or facilitate each other, from the perspective of patients and clinicians, is key to informing clinical, health services and patient-management interventions to improve diabetes management during cancer treatments. In a survey of people with diabetes (n=37), Hershey et al. [44,45] found that patient-reported reductions in diabetes self-management activities during cancer chemotherapy were associated with greater symptom burden and lower diabetes self-efficacy. Hershey et al.'s survey also included two open-ended questions about the impact of cancer on diabetes, which revealed that many patients prioritised cancer care over diabetes care, with some reporting advice from primary-care providers not to be concerned with diabetes during chemotherapy. In a focus-group with patients (n=5), Hershey et al. [46] similarly found that patients reported that cancer treatment took priority.

Hershey and colleagues also conducted focus-groups with oncology clinicians (n=20) [46], finding that oncologists generally saw diabetes management to be outside their remit, and the responsibility of primary-care, but noted poor communication between oncology and primary-care. These findings were recently corroborated by Cho et al. [47], who interviewed oncologists (n=10) and primary-care doctors and nurses (n=10) about diabetes management during cancer treatment. Cho et al. found both oncology and primary-care providers thought primary-care should be responsible for diabetes care, though noted barriers to this including very infrequent and limited communication between oncology and primary-care, and the fact that many patients reduce contact with primary-care following a cancer diagnosis.

Though these qualitative studies provide important insights into the challenges of managing diabetes during cancer treatments, they have involved a relatively small number of patients and clinicians, and are limited in scope and depth. Hershey's studies with patients [44-46] both focused on only chemotherapy cancer treatment, and predominately surveyed participants during this treatment, meaning they could not obtain perspectives on other elements of cancer treatment (e.g., radiotherapy, long-term tamoxifen) or with the benefit of reflection (i.e., looking back on completed treatment). Furthermore, with just two open-ended questions, Hershey et al.'s survey study did not

For peer review only

undertake an in-depth examination of participants' experiences. The studies with clinicians by Hershey [46] and Cho [47] both focused exclusively on T2DM and do not include the perspectives of several professions relevant to diabetes management during cancer, including diabetes doctors and specialist nurses, anaesthetists, and dieticians. Moreover, these prior studies have focused on barriers to diabetes management during cancer treatments, with limited or no focus on patients' and clinicians' perspectives on enabling factors and potential interventions. Also, current studies are exclusively USA-based, and findings may to some degree be context-specific, given differences in the organisation and financing of heath care systems globally, even within higher-income countries. We aim to extend, and address some of the limitations of, this previous qualitative work.

Our study aims to extend previous qualitative work in this area and help inform intervention development

The current qualitative interview study aims to identify and elucidate challenges and enablers to diabetes management and control during treatment for cancer, based on the experiences and perspectives of people with diabetes and comorbid cancer and health care professionals involved in their care. To facilitate examination of a comprehensive range of individual and service level barriers and enablers, and to support the identification of potential pertinent intervention approaches to address and harness these respectively, we will use the Theoretical Domains Framework (TDF) [48,49], and related Theory and Techniques Tool (TTT) [50-53] and Behaviour Change Wheel (BCW) [54,55], to inform data collection and analysis, as detailed in the Methods section.

Research questions

1. What are the patient-perceived challenges and enablers to effective self-management and control of diabetes during cancer treatments?

2. What are the clinician-perceived challenges and enablers to effective clinical management and control of diabetes during cancer treatments?

3. What are patients' suggestions for ways to support and improve self-management and control of diabetes during cancer treatments?

4. What are clinicians' suggestions for ways to support and improve clinical management and control of diabetes during cancer treatments?

5. What are potentially promising intervention targets and strategies for consideration in future research to optimise patient and/or clinician management and control of diabetes during cancer treatments?

For peer review only

METHODS AND ANALYSIS

Participants

Eligibility criteria are detailed in Table 1.

Table 1 Participant eligibility criteria

	Inclusion	*Exclusion*
Patients	➢ Medically-diagnosed type 1 or type 2 diabetes<br>➢ Subsequent diagnosis of breast, prostate, or colorectal cancer (in the UK, three of the four most common cancers and the largest survivor groups [56])<br>➢ Received any type of localised or systemic National Health Service anti-cancer treatment (currently or within the last 3-years)	➢Under 18-years of age<br>➢ Clinician-estimated life expectancy of less than 3-months<br>➢ Lack capacity to provide informed consent
Clinicians	➢ Involved in providing care to above comorbid patient group (i.e., cancer patients with pre-existing diabetes) in relation to their diabetes and/or cancer	

Recruitment sites and procedures

Patients

Hospital-based recruitment

Patients will be primarily recruited from Cancer Centres in two Yorkshire-based National Health Service (NHS) Hospital Trusts. Clinical teams, possibly with Clinical Research Network support, will identify eligible patients and first approach them about the study, providing a patient information sheet. Interested patients will contact our research team directly, or, if a patient requests it, the clinical team will pass onto us patient-provided contact information and we will initiate correspondence.

Community-advertisement recruitment

We will also recruit patients via an advertisement flyer calling for people aged 18+ "with diabetes (type 1 or type 2), who are being treated for breast, bowel or prostate cancer, or have been in the

For peer review only

past 3 years" to "tell us about your experiences of managing diabetes during cancer treatments". The flyer will be disseminated via social media (e.g., Twitter and Facebook accounts of the research team members), and by relevant willing UK-based charities and organisations (e.g., in newsletters). Patients who see the flyer and are interested will contact our research team directly.

Clinicians

Hospital-based recruitment

Clinicians will be recruited from the Cancer Centres, as well as other relevant hospital departments and specialties (e.g., endocrinology, anaesthesia, pharmacy, dietetics) within the participating hospitals. Our research team includes oncology and diabetes clinicians working at the participating hospital Trusts. Potentially eligible clinicians will be identified and emailed a staff information sheet by a member of the research team, or by a gatekeeper colleague who has agreed to disseminate study information. Eligible and interested clinicians contact the research team directly.

Primary-care recruitment

We will also recruit General Practitioners (GPs) and practice nurses working at general practices in Yorkshire. GPs and practice nurses will be informed about the study by local primary-care R&D teams who are willing to disseminate study information (e.g., in a CCG-newsletter), or will be emailed a staff information sheet by a member of the research team (e.g., GPs with a part-time academic post known to the research team). Eligible and interested clinicians contact the research team directly.

Sample size and sampling strategy

We will recruit and interview 30-40 patients and 30-40 clinicians. Based on our experience, this sample size will enable adequate sample diversity on key participant characteristics and allow us to reach sufficient data saturation.

We aim to recruit comparable numbers of people with breast, prostate, and colorectal cancer (n≈10-13 each cancer type), with both diabetes types represented in each cancer subgroup (2-3 patients in each cancer subgroup with type 1 diabetes, which accounts for 7-12% of all diabetes cases in high-income countries [1]). We also aim for some diversity in the sample as a whole with regard to types of cancer treatment, sociodemographic characteristics (gender, age, ethnicity), and the extent of experienced difficulties with diabetes management during cancer treatments.

We aim to recruit clinicians from a wide range of relevant professions and specialities (e.g., oncologists, radiographers, surgeons, diabetologists, specialist nurses, dieticians, GPs) and for some diversity in the sample as a whole with regard to professional seniority.

The composition of the sample will be monitored during recruitment and, if necessary and possible, targeted recruitment of under-represented groups will be undertaken.

Theoretical framework informing data collection and analysis

We will use the TDF [48,49] and related resources to inform data collection and analysis. The TDF synthesises key constructs in numerous theories of behaviour and behaviour change, and thus

For peer review only

"provides a theoretical lens through which to view the cognitive, affective, social and environmental influences on behaviour" [p.2,57]. The TDF version-2 comprises 84 constructs theorised to influence behaviour (e.g., professional identity; self-efficacy; cognitive overload/tiredness) organised by 14 domains (e.g., social/professional role and identity; beliefs about consequences; environmental context and resources). These domains can be considered Mechanisms of Action (MoA), that is processes which influence behaviour, and are thus potential intervention targets.

The TDF is part of an evolving set of resources being developed by Michie et al. to promote design of more effective theory-based behavioural interventions. These resources include the TTT [50-53], which provides guidance on linking MoAs to pertinent Behaviour Change Techniques (BCTs) (e.g., verbal persuasion about capability; conserving mental resources; information about health consequences), which are the potentially 'active ingredients' in an intervention that changes behaviour. The TTT synthesises the evidence (or not) for links between 74 BCTs and 26 MoAs, which include the 14 TDF domains and the 12 most frequently occurring MoAs which did not overlap with these identified in a review of behaviour change theories [52]. The BCW [54,55] is a 'theory and evidence based' intervention development approach [58] that provides guidance on considering and identifying the function of interventions (e.g., education, persuasion, enablement) and policy categories that may support the delivery of these functions (e.g., guidelines, service provision, environmental planning) and how these both link to MoAs and/or BCTs.

The TDF and related resources have been used to inform the development and evaluation of health-focused behavioural interventions [e.g.,59-61], including informing the data collection and/or analysis of qualitative studies forming an early stage in the process of intervention development [62-65].

Data collection: interview content and procedures

Participants will take part in one semi-structured qualitative interview lasting around 45 minutes, though duration is likely to be variable depending on how much a participant wishes to say and their preferred interview pace. Participants can choose their interview date/time and mode (e.g., telephone, videocall, in person) provided arrangements adhere to current relevant government and workplace rules around COVID-19 social distancing.

Interviews will examine participants' experiences of and perspectives on diabetes management and control during cancer treatments. Early interview questions will enquire about key sociodemographic characteristics and relevant clinical (e.g., diabetes and cancer type and treatments) or professional (e.g., job-title, workplace) details. Interviews will seek to identify and elucidate patient- and clinician-perceived challenges and enablers to effective diabetes management and glycemic control in the context of cancer diagnosis and treatment, and ways to address and optimise these respectively.

Interview guides were developed, informed by: (1) previous research [e.g.,44-47]; (2) advice and feedback from the study PPI and steering groups; and lastly (3) the MoA covered by the TTT [50] which, as previously discussed, includes the TDF domains. We were mindful interviews do not become dominated by examining MoAs, especially given multiple behaviours are involved in managing diabetes (e.g., prescribing medications, following dietary advice, blood glucose self-monitoring) and to systematically examine each MoA in relation to each different behaviour that may be discussed would make for an overly long, repetitive, and granular-level interview. Also, we did not want interviews to be restricted to examining only the MoAs, thereby potentially overlooking other influential factors. Thus, in an approach consistent with recent recommendations for using the

For peer review only

TDF in qualitative studies [66], the interview guides contained at least one question or follow-up question likely to encourage discussion of challenges or enablers relevant to each MoA, rather than a highly-structured list of MoA-focused questions, one per MoA per different behaviours. Pilot interviews were undertaken with 3 clinician co-applicants and 3 PPI-group members, to refine the interview guides and hone interviewer technique. Box 1 shows sample questions from the patient and clinician interview guides. Interviews will be audio-recorded.

Box 1 Sample interview questions from the interview guides

Patient interviews

Selected opening questions to key topic areas and example follow-up questions

➢ In what ways, if any, did your diabetes management change during the time that you were/are having cancer treatment?
  – any changes to the foods you ate?
➢ In terms of managing your diabetes during cancer treatments, what have you found to be your biggest challenges?
  – has it been difficult to remember (e.g., to take tablets, self-monitor blood glucose) during treatment?
➢ How important do you feel it is to effectively manage your diabetes throughout cancer treatments?
  – is it more or less important to you than before cancer?
➢ Did you receive any information about diabetes management during cancer treatments?
  – from who, when?
➢ What has the support been like from health care professionals in terms of managing your diabetes during the time that you were having cancer treatment?
  – how important did it seem to your (e.g., oncologist, GP) to manage your diabetes well during this time?
➢ Have any family or friends been involved in helping you to manage your diabetes during the time you were having cancer treatment?
  – how do they help?
➢ What do you think could be done to help people to manage their diabetes better during the time they are having cancer treatment?
  – why would that help?

Example cross-topic follow-up questions

- is that something you know about?
- is that something you know how to do?
- is that something that would be easy or difficult to do?
- what would prompt you to do that?
- what would be the reasons to do /not do that?
- and do you think your family and friends / doctors tend to think similarly?
- what would help you with that?

For peer review only

Clinician interviews

Selected opening questions to key topic areas and example follow-up questions

➢ As a (e.g., medical oncologist, GP), what do you see as your role in supporting clinical management of diabetes during cancer treatments?

– and in supporting patients' self-management of their diabetes?

➢ Can you tell me about how you identify this patient group – that you know you're dealing with a patient who has both cancer *and* diabetes

– once you know a patient has diabetes/is having cancer treatment, would it cause you to do anything differently?

➢ How important do you feel it is to effectively manage diabetes during a time that someone is also having cancer treatments?

– what do you see are the benefits of good / risks of poor diabetes management during cancer treatments?

➢ Are there any care protocols or clinical guidelines you follow when caring and making decisions for this group?

– do you find these useful?

➢ Do patients receive any information about or support with diabetes management during cancer treatments?

– do patients tend to ask for information/help?

➢ If there are difficulties controlling a patients' diabetes during cancer treatment what sorts of things would be done to address this?

– what about altering medications / bringing other professionals in / trying to improve patient self-management?

➢ Thinking of your work environment, what improvements would make it easier for you to support good diabetes management for this group?

– what about resources / recording and reporting systems / workflows and processes / culture?

Example cross-topic follow-up questions

- is that something you know how to do?
- is that something that would be easy or difficult to do?
- is that something you feel confident to do?
- is that something you always/usually/often do?
- how would you know if that had been done?
- and do you think your colleagues tend to think/do similarly?
- what would need to happen for you to do that / be able to do that?

Data analysis

Primary

The interview data will be analysed to identify and elucidate patient and clinician perceived challenges and enablers to self and clinical diabetes management and control during cancer treatments, and suggested ways to overcome and optimise these respectively. We will use the Framework method [67], guided by the stages and recommendations set out by Gale et al. [68]: (1)

For peer review only

verbatim transcription; (2) familiarisation with the data; (3) coding; (4) developing a working analytical framework; (5) applying the framework; (6) charting data into a framework matrix (a summary of the data by analytic code/category per participant); and (7) interpreting the data.

Analysis will overlap with data collection. To ensure rigour, analysis will be an iterative, collaborative process led by the core research team members (LA,IK,LK,MP,JT), who have experience of framework analysis and using the TDF in qualitative research [e.g.,63,69,70], with input and feedback from other members of the research team and the PPI and steering groups at key points. We will use QSR-NVivo software to support analysis, and document substantive decisions during the analytic process.

Coding will use both deductive codes (i.e., based on the research questions, interview guides) and inductive codes (i.e., based on reading a sample of transcripts), which will be generated through iterative rounds of independent work and subsequent group discussion. In line with recent recommendations for using the TDF in qualitative studies [66], we do not intend to include the MoAs as *a priori* codes, but to subsequently consider inductively-generated findings about challenges and enablers against the MoAs (see below). This will guard against overlooking challenges and enablers discussed in the interviews not covered by the TTT and, as the TTT includes a substantial number of MoAs (n=26), guard against other key focuses of the analysis being overshadowed (e.g., interviewees suggestions for tackling challenges).

A working analytical framework will be iteratively developed through agreeing by consensus upon a final set of codes, and their initial organisation into categories, informed by input from the PPI-group and wider research team. Members of the PPI-group will each read a different sample of transcripts and provide coding-relevant feedback (e.g., feedback on sections that stood out to them, on barriers and enablers discussed in the interview). Other members of the research team and steering-group will be asked to review and feedback on a draft(s) of the working analytical framework.

The analytical framework will be applied to all interview transcripts, and subsequently the data will be charted into a framework matrix, including references to illustrative quotations. A proportion of transcripts will be double-coded and double-charted (≈10% at both stages), and compared and discussed, to ensure consistent application of the framework and data charting. We will identify and develop themes and sub-themes pertinent to the research questions, using the matrix to facilitate comparison within and between codes/categories and participants, and thus the identification of patterns and deviant cases in the data, including on the basis of diabetes and/or cancer type and/or treatments. Members of the PPI-group, wider research team and steering-group will input into data interpretation by reviewing and providing feedback on the matrix and a related working draft(s) of themes and sub-themes.

We envisage the patient and clinician data will initially be coded and charted separately, but subsequently synthesised as much as possible during interpretation and theme development. However, decisions about this aspect of the analysis will be made during analysis, after familiarisation with the data.

Secondary

In a second phase of analysis we will: (i) map the challenges and enablers identified in the framework analysis phase to the MoAs; (ii) prioritise MoAs for targeting in interventions; and (iii) determine pertinent intervention functions and BCTs to deliver these. We will map to the MoAs using current definitions (e.g.,[50]), and by independent work followed by group discussion to

For peer review only

achieve a consensus. Target MoAs will be prioritised through consensus discussion, on the basis of the mapping results, the contextualised understanding of barriers and ways to address these provided by the framework analysis of the interview data, and views of the study PPI-group and wider research team and steering-group (which include multidisciplinary clinicians). For each prioritised MoA, we will determine, through consensus discussion, potential intervention functions and policy categories using the BCW [54,55] and pertinent BCTs using the TTT [50]. These processes are consistent with those undertaken in previous qualitative studies, including by members of our team, which have similarly sought to understand, and inform interventions to address, health care challenges (e.g., gestational diabetes [62]; deprescribing [63]; smoking in pregnancy [65]).

Patient and Public involvement (PPI)

Six PPI-representatives, with personal experience of diabetes, cancer or both conditions as a patient or carer, were involved in developing this research and helped shape the design and methods of the study. One of these PPI-representatives was a co-applicant on our grant application for funding and is a co-author of this protocol paper.

We have established a study PPI-group with 6 members, one of whom, co-applicant and protocol co-author MM, was involved in the previous study development stage. As well as PPI-group meetings, MM will also attend study steering-group meetings, helping ensure effective communication between these two groups. The PPI-group will collaborate, advise and feedback on all stages of the research including: design and piloting of the interview guides; data analysis including coding and interpreting the data; and dissemination outputs including lay summaries, presentations and journal papers. We will discuss with PPI-group members the research activities they wish to be involved in, their relevant prior experience (if any), and therefore what training they may require (e.g., practising data coding using transcripts from the pilot interviews), and arrange as and when appropriate. We will be guided by the UK Standards for Public Involvement [71], and PPI-representatives will be paid following UK National Institute for Health Research guidance [72] and reimbursed any expenses.

ETHICS AND DISSEMINATION

Approvals and ethical considerations

The study has approval from the NHS West Midlands – Edgbaston Research Ethics Committee (20/WM/0310), NHS Health Research Authority (IRAS-ID:276694) and the Leeds Beckett University Psychology Research Ethics Committee.

Participants will receive written study information which will include contact details of organisations providing diabetes- and cancer-related information and support (e.g., Macmillan helpline). Consent will be obtained in writing or verbally, depending on participant preference; verbal consent will be recorded immediately prior to the interview and stored on a separate audio-file to the interview. Participants will be informed of their right to choose, without needing to provide a reason why, to not answer an interview question(s), to take a break or stop the interview at any time, and to request withdrawal of any/all of their data. Research and personal data will be handled and stored confidentially and securely in line with government and Leeds Beckett University data protection requirements and guidelines. We will protect participant anonymity and make

For peer review only

unidentifiable all illustrative quotes used in reports of the findings. Participants will not be offered any incentives.

Dissemination

Findings will be presented to lay, clinical, academic and NHS and charity service-provider audiences via dissemination of written summaries and articles, infographics, and presentations. Findings will be published in a peer-reviewed journal(s) following COREQ and SRQR reporting-guidelines [73,74]. Study data may be made available on reasonable request to the corresponding author.

This UK-based study will be the first published study of its kind outside the USA, and will provide the most in-depth qualitative examination of this topic to date, with a focus on both barriers and enablers, and ways to address and harness these respectively, for both patient and clinician diabetes management during cancer treatments. Our study will extend previous work by also interviewing a wider range of clinicians, crucially including diabetologists, and considering other cancer treatments in addition to chemotherapy. Furthermore, whilst previous work in this area has not been theoretically-informed, at least explicitly, we will use the TDF, TTT, and BCW [48-55] to facilitate examination of a comprehensive range of barriers and enablers and support identification of pertinent and feasible intervention approaches.

There is increasing interest worldwide in improving glycemic control during cancer treatments, evident in the recent development of interventions for people with diabetes having treatment for cancer, such as clinical guidelines in UK [75], new integrated care pathways in Italy [76], and a clinical pharmacy intervention and counselling program in Turkey [77], though intervention research in this area is in its infancy. Findings of this study will be used to inform development and implementation of clinical, health services and patient-management intervention strategies to optimise diabetes management and control during cancer treatments.

Timeline

Data collection commenced February-2021 and is projected to close August-2022.

Contributors

LA conceived the study, led on its development and design, and is Chief Investigator and lead applicant of the grant funding this study; SK, IK, LK, FM, MM, DS, JT, GV, & JW contributed to the development of the study design and protocol and are co-applicants of the funding grant; MP is the appointed study research assistant and will undertake the interviews; the roles of team members in analysis are specified in the manuscript; LA drafted this manuscript and all co-authors reviewed, provided feedback, and approved it for submission.

For peer review only

Funding

This study is funded by the National Institute for Health Research (NIHR) Research for Patient Benefit programme, Award ID: NIHR201179. The views expressed are those of the author(s) and not necessarily those of the NIHR or the Department of Health and Social Care.

Competing interests

None

References

1. Ogurtsova K, da Rocha Fernandes JD, Huang Y, et al. IDF Diabetes Atlas: Global estimates for the prevalence of diabetes for 2015 and 2040. *Diabetes Res Clin Pract* 2017;128:40-50. doi: 10.1016/j.diabres.2017.03.024.
2. Saeedi P, Petersohn I, Salpea P, et al. Global and regional diabetes prevalence estimates for 2019 and projections for 2030 and 2045: Results from the International Diabetes Federation Diabetes Atlas, 9th edition. *Diabetes Res Clin Pract* 2019;157:107843. doi: 10.1016/j.diabres.2019.107843.
3. www.diabetes.org.uk/about_us/news/diabetes-prevalence-2019 (accessed Dec 2021)
4. Harding JL, Shaw JE, Peeters A, et al. Cancer risk among people with type 1 and type 2 diabetes: disentangling true associations, detection bias, and reverse causation. *Diabetes Care* 2015;38(2):264-70. doi: 10.2337/dc14-1996.
5. Lega IC, Lipscombe LL. Review: Diabetes, obesity, and cancer-pathophysiology and clinical implications. *Endocr Rev* 2020;41(1):bnz014. doi: 10.1210/endrev/bnz014.
6. Ling S, Brown K, Miksza JK, et al. Risk of cancer incidence and mortality associated with diabetes: A systematic review with trend analysis of 203 cohorts. *Nutr Metab Cardiovasc Dis* 2021;31(1):14-22. doi: 10.1016/j.numecd.2020.09.023.
7. Marley J, Nicholl BI, Macdonald S, et al. Associations between long-term conditions and upper gastrointestinal cancer incidence: A prospective population-based cohort of UK Biobank participants. *J Comorb* 2021;11:26335565211056136. doi: 10.1177/26335565211056136.
8. Ahmad AS, Ormiston-Smith N, Sasieni PD. Trends in the lifetime risk of developing cancer in Great Britain: comparison of risk for those born from 1930 to 1960. *Br J Cancer* 2015;112(5):943-7. doi: 10.1038/bjc.2014.606.
9. van de Poll-Franse LV, Houterman S, Janssen-Heijnen ML, et al. Less aggressive treatment and worse overall survival in cancer patients with diabetes: a large population based analysis. *Int J Cancer* 2007;120(9):1986-92. doi: 10.1002/ijc.22532.
10. Louwman WJ, Aarts MJ, Houterman S, et al. A 50% higher prevalence of life-shortening chronic conditions among cancer patients with low socioeconomic status. *Br J Cancer* 2010;103(11):1742-8. doi: 10.1038/sj.bjc.6605949.
11. Koo MM, Swann R, McPhail S, et al. The prevalence of chronic conditions in patients diagnosed with one of 29 common and rarer cancers: A cross-sectional study using primary care data. *Cancer Epidemiol* 2020;69:101845. doi: 10.1016/j.canep.2020.101845.

12. Fowler H, Belot A, Ellis L, et al. Comorbidity prevalence among cancer patients: a population-based cohort study of four cancers. *BMC Cancer* 2020;20(1):2. doi: 10.1186/s12885-019-6472-9.
13. Stark Casagrande S, Fradkin JE, Saydah SH, et al. The prevalence of meeting A1C, blood pressure, and LDL goals among people with diabetes, 1988-2010. *Diabetes Care* 2013;36(8):2271-9. doi: 10.2337/dc12-2258.
14. Blonde L, Aschner P, Bailey C, et al. Gaps and barriers in the control of blood glucose in people with type 2 diabetes. *Diab Vasc Dis Res* 2017;14(3):172-83. doi: 10.1177/1479164116679775.
15. Bultz BD, Carlson LE. Emotional distress: the sixth vital sign--future directions in cancer care. *Psychooncology* 2006;15(2):93-5. doi: 10.1002/pon.1022.
16. Zhu J, Fang F, Sjölander A, et al. First-onset mental disorders after cancer diagnosis and cancer-specific mortality: a nationwide cohort study. *Ann Oncol* 2017;28(8):1964-9. doi: 10.1093/annonc/mdx265.
17. Carlson LE, Zelinski EL, Toivonen KI, et al. Prevalence of psychosocial distress in cancer patients across 55 North American cancer centers. *J Psychosoc Oncol* 2019;37(1):5-21. doi: 10.1080/07347332.2018.1521490.
18. Pearce A, Haas M, Viney R, et al. Incidence and severity of self-reported chemotherapy side effects in routine care: A prospective cohort study. *PLoS One* 2017;12(10):e0184360. doi: 10.1371/journal.pone.0184360.
19. García-Chías B, Figuero E, Castelo-Fernández B, et al. Prevalence of oral side effects of chemotherapy and its relationship with periodontal risk: a cross sectional study. *Support Care Cancer* 2019;27(9):3479-90. doi: 10.1007/s00520-019-4650-6.
20. Pettit S, Cresta E, Winkley K, et al. Glycaemic control in people with type 2 diabetes mellitus during and after cancer treatment: A systematic review and meta-analysis. *PLoS One* 2017;12(5):e0176941. doi: 10.1371/journal.pone.0176941.
21. Pinheiro LC, Kaur H, Nilo D, et al. Determining the impact of a cancer diagnosis on diabetes management: a systematic literature review. *Am J Clin Oncol* 2019;42(11):870-83. doi: 10.1097/COC.0000000000000612.
22. Yao N, Camacho FT, Chukmaitov AS, et al. Diabetes management before and after cancer diagnosis: missed opportunity. *Ann Transl Med* 2015;3(5):72. doi: 10.3978/j.issn.2305-5839.2015.03.52.
23. Pinheiro LC, Soroka O, Kern LM, et al. Diabetes care management patterns before and after a cancer diagnosis: A SEER-Medicare matched cohort study. *Cancer* 2020;126(8):1727-35. doi: 10.1002/cncr.32728.
24. Pinheiro LC, Soroka O, Kern LM, et al. Racial disparities in diabetes care among incident breast, prostate, and colorectal cancer survivors: a SEER Medicare study. *J Cancer Surviv* 2021 Mar 4. doi: 10.1007/s11764-021-01003-z. Epub ahead of print.
25. Srokowski TP, Fang S, Hortobagyi GN, et al. Impact of diabetes mellitus on complications and outcomes of adjuvant chemotherapy in older patients with breast cancer. *J Clin Oncol* 2009;27(13):2170-6. doi: 10.1200/JCO.2008.17.5935.
26. Zaorsky NG, Shaikh T, Ruth K, et al. Prostate cancer patients with unmanaged diabetes or receiving insulin experience inferior outcomes and toxicities after treatment with radiation therapy. *Clin Genitourin Cancer* 2017;15(2):326-35.e3. doi: 10.1016/j.clgc.2016.08.020.

27. Stein KB, Snyder CF, Barone BB, et al. Colorectal cancer outcomes, recurrence, and complications in persons with and without diabetes mellitus: a systematic review and meta-analysis. *Dig Dis Sci* 2010;55(7):1839-51. doi: 10.1007/s10620-009-0944-8.
28. Iavazzo C, McComiskey M, Datta M, et al. Preoperative HBA1c and risk of postoperative complications in patients with gynaecological cancer. *Arch Gynecol Obstet* 2016;294(1):161-4. doi: 10.1007/s00404-015-3983-8.
29. Lopez-de-Andres A, Jimenez-Trujillo I, Hernandez-Barrera V, et al. Association of type 2 diabetes with in-hospital complications among women undergoing breast cancer surgical procedures. A retrospective study using the Spanish National Hospital Discharge Database, 2013-2014. *BMJ Open* 2017;7(11):e017676. doi: 10.1136/bmjopen-2017-017676.
30. Zylla D, Gilmore G, Eklund J, et al. Impact of diabetes and hyperglycemia on health care utilization, infection risk, and survival in patients with cancer receiving glucocorticoids with chemotherapy. *J Diabetes Complications* 2019;33(4):335-9. doi: 10.1016/j.jdiacomp.2018.12.012.
31. Caputo MP, Shabani S, Mhaskar R, et al. Diabetes mellitus in major head and neck cancer surgery: systematic review and meta-analysis. *Head Neck* 2020;42(10):3031-40. doi: 10.1002/hed.26349.
32. Barone BB, Yeh HC, Snyder CF, et al. Postoperative mortality in cancer patients with preexisting diabetes: systematic review and meta-analysis. *Diabetes Care* 2010;33(4):931-9. doi: 10.2337/dc09-1721.
33. Harding JL, Andes LJ, Gregg EW, et al. Trends in cancer mortality among people with vs without diabetes in the USA, 1988-2015. *Diabetologia* 2020;63(1):75-84. doi: 10.1007/s00125-019-04991-x.
34. Jing C, Wang Z, Fu X. Effect of diabetes mellitus on survival in patients with gallbladder Cancer: a systematic review and meta-analysis. *BMC Cancer* 2020;20(1):689. doi: 10.1186/s12885-020-07139-y.
35. Jackson RS, Amdur RL, White JC, et al. Hyperglycemia is associated with increased risk of morbidity and mortality after colectomy for cancer. *J Am Coll Surg* 2012;214(1):68-80. doi: 10.1016/j.jamcollsurg.2011.09.016.
36. Kang SG, Hwang EC, Jung SI, et al. Poor preoperative glycemic control is associated with dismal prognosis after radical nephroureterectomy for upper tract urothelial carcinoma: a Korean multicenter study. *Cancer Res Treat* 2016;48(4):1293-301. doi: 10.4143/crt.2016.021.
37. Li J, Ning NY, Rao QX, et al. Pretreatment glycemic control status is an independent prognostic factor for cervical cancer patients receiving neoadjuvant chemotherapy for locally advanced disease. *BMC Cancer* 2017;17(1):517. doi: 10.1186/s12885-017-3510-3.
38. Nik-Ahd F, Howard LE, Eisenberg AT, et al. Poorly controlled diabetes increases the risk of metastases and castration-resistant prostate cancer in men undergoing radical prostatectomy: Results from the SEARCH database. *Cancer* 2019;125(16):2861-7. doi: 10.1002/cncr.32141.
39. Liang SH, Shen YC, Wu JY, et al. Impact of poor preoperative glycemic control on outcomes among patients with cervical cancer undergoing a radical hysterectomy. *Oncol Res Treat* 2020;43(1-2):10-8. doi: 10.1159/000502911.
40. Zeng X, Xu C, Cheng J, et al. Poor glycemic control might compromise the efficacy of chemotherapy in non-small cell lung cancer patients with diabetes mellitus. *Cancer Med* 2020;9(3):902-11. doi: 10.1002/cam4.2750.

41. Hershey DS, Hession S. Chemotherapy and glycemic control in patients with type 2 diabetes and cancer: a comparative case analysis. *Asia Pac J Oncol Nurs* 2017;4(3):224-32. doi: 10.4103/apjon.apjon_22_17.
42. Lavdaniti M, Michalopoulou S, Owens DA, et al. The impact of comorbid diabetes type 2 on quality of life in cancer patients undergoing chemotherapy. *Endocr Metab Immune Disord Drug Targets* 2021;21(6):1017-24. doi: 10.2174/1871530320666200729151715.
43. Worndl E, Fung K, Fischer HD, et al. Preventable diabetic complications after a cancer diagnosis in patients with diabetes: a population-based cohort study. *JNCI Cancer Spectr* 2018;2(1):pky008. doi: 10.1093/jncics/pky008.
44. Hershey DS, Tipton J, Given B, et al. Perceived impact of cancer treatment on diabetes self-management. *Diabetes Educ* 2012;38(6):779-90. doi: 10.1177/0145721712458835.
45. Hershey DS, Given B, Given C, et al. Predictors of diabetes self-management in older adults receiving chemotherapy. *Cancer Nurs* 2014;37(2):97-105. doi: 10.1097/NCC.0b013e3182888b14.
46. Goebel J, Valinski S, Hershey DS. Improving coordination of care among healthcare professionals and patients with diabetes and cancer. *Clin J Oncol Nurs* 2016;20(6):645-651. doi: 10.1188/16.CJON.645-651.
47. Cho J, Nilo D, Sterling MR, et al. Eliciting primary care and oncology provider perspectives on diabetes management during active cancer treatment. *Support Care Cancer* 2021;29(11):6881-90. doi: 10.1007/s00520-021-06264-z.
48. Michie S, Johnston M, Abraham C, et al. Making psychological theory useful for implementing evidence based practice: a consensus approach. *Qual Saf Health Care* 2005;14(1):26-33. doi: 10.1136/qshc.2004.011155.
49. Cane J, O'Connor D, Michie S. Validation of the theoretical domains framework for use in behaviour change and implementation research. *Implement Sci* 2012;7:37. doi: 10.1186/1748-5908-7-37.
50. theoryandtechniquetool.humanbehaviourchange.org/ (accessed Dec 2021)
51. Carey RN, Connell LE, Johnston M, et al. Behavior change techniques and their mechanisms of action: a synthesis of links described in published intervention literature. *Ann Behav Med* 2019;53(8):693-707. doi: 10.1093/abm/kay078.
52. Connell LE, Carey RN, de Bruin M, et al. Links between behavior change techniques and mechanisms of action: an expert consensus study. *Ann Behav Med* 2019;53(8):708-20. doi: 10.1093/abm/kay082.
53. Johnston M, Carey RN, Connell Bohlen LE, et al. Development of an online tool for linking behavior change techniques and mechanisms of action based on triangulation of findings from literature synthesis and expert consensus. *Transl Behav Med* 2021;11(5):1049-65. doi: 10.1093/tbm/ibaa050.
54. Michie S, van Stralen MM, West R. The behaviour change wheel: a new method for characterising and designing behaviour change interventions. *Implement Sci* 2011;6:42. doi: 10.1186/1748-5908-6-42.
55. Michie S, Atkins L, West R. The Behaviour Change Wheel: a guide to designing interventions. London: Silverback Publishing 2014.
56. Maddams J, Utley M, Møller H. Projections of cancer prevalence in the United Kingdom, 2010-2040. *Br J Cancer* 2012;107(7):1195-202. doi: 10.1038/bjc.2012.366.

For peer review only - http://bmjopen.bmj.com/site/about/guidelines.xhtml

57. Atkins L, Francis J, Islam R, et al. A guide to using the Theoretical Domains Framework of behaviour change to investigate implementation problems. *Implement Sci* 2017;12(1):77. doi: 10.1186/s13012-017-0605-9.
58. O'Cathain A, Croot L, Sworn K, et al. Taxonomy of approaches to developing interventions to improve health: a systematic methods overview. *Pilot Feasibility Stud* 2019;5:41. doi: 10.1186/s40814-019-0425-6.
59. Craig LE, Taylor N, Grimley R, et al. Development of a theory-informed implementation intervention to improve the triage, treatment and transfer of stroke patients in emergency departments using the Theoretical Domains Framework (TDF): the T^3 Trial. *Implement Sci* 2017;12(1):88. doi: 10.1186/s13012-017-0616-6.
60. Fahim C, Acai A, McConnell MM, et al. Use of the theoretical domains framework and behaviour change wheel to develop a novel intervention to improve the quality of multidisciplinary cancer conference decision-making. *BMC Health Serv Res* 2020;20(1):578. doi: 10.1186/s12913-020-05255-w.
61. Reidy C, Foster C, Rogers A. A facilitated web-based self-management tool for people with type 1 diabetes using an insulin pump: intervention development using the behavior change wheel and theoretical domains framework. *J Med Internet Res* 2020;22(5):e13980. doi: 10.2196/13980.
62. Martis R, Brown J, McAra-Couper J, et al. Enablers and barriers for women with gestational diabetes mellitus to achieve optimal glycaemic control - a qualitative study using the theoretical domains framework. *BMC Pregnancy Childbirth* 2018;18(1):91. doi: 10.1186/s12884-018-1710-8.
63. Scott S, Twigg MJ, Clark A, et al. Development of a hospital deprescribing implementation framework: A focus group study with geriatricians and pharmacists. *Age Ageing* 2019;49(1):102-10. doi: 10.1093/ageing/afz133.
64. Maharaj AD, Evans SM, Zalcberg JR, et al. Barriers and enablers to the implementation of multidisciplinary team meetings: a qualitative study using the theoretical domains framework. *BMJ Qual Saf* 2021;30(10):792-803. doi: 10.1136/bmjqs-2020-011793.
65. Griffiths SE, Naughton F, Brown KE. Accessing specialist support to stop smoking in pregnancy: A qualitative study exploring engagement with UK-based stop smoking services. *Br J Health Psychol* 2021 Dec 1. doi: 10.1111/bjhp.12574. Epub ahead of print.
66. McGowan LJ, Powell R, French DP. How can use of the Theoretical Domains Framework be optimized in qualitative research? A rapid systematic review. *Br J Health Psychol* 2020;25(3):677-94. doi: 10.1111/bjhp.12437.
67. Ritchie J, Lewis J, McNaughton Nicholls C, et al. Qualitative research practice: a guide for social science students and researchers (2 ed.). London: Sage 2014.
68. Gale NK, Heath G, Cameron E, et al. Using the framework method for the analysis of qualitative data in multi-disciplinary health research. *BMC Med Res Methodol* 2013;13:117. doi: 10.1186/1471-2288-13-117.
69. Schnabel S, van Wijck F, Bain B, et al. Experiences of augmented arm rehabilitation including supported self-management after stroke: a qualitative investigation. *Clin Rehabil* 2021;35(2):288-301. doi: 10.1177/0269215520956388.
70. Ahern S, Arnott B, Chatterton T, et al. Understanding parents' school travel choices: a qualitative study using the Theoretical Domains Framework. *Journal of Transport and Health* 2017;4:278-93. doi: 10.1016/j.jth.2016.11.001

71. www.invo.org.uk/wp-content/uploads/2019/11/UK-standards-for-public-involvement-v6.pdf (accessed Dec 2021)
72. www.nihr.ac.uk/documents/payment-guidance-for-researchers-and-professionals/27392 (accessed Dec 2021)
73. Tong A, Sainsbury P, Craig J. Consolidated criteria for reporting qualitative research (COREQ): a 32-item checklist for interviews and focus groups. *Int J Qual Health Care* 2007;19(6):349-57. doi: 10.1093/intqhc/mzm042.
74. O'Brien BC, Harris IB, Beckman TJ, et al. Standards for reporting qualitative research: a synthesis of recommendations. *Acad Med* 2014;89(9):1245-51. doi: 10.1097/ACM.0000000000000388.
75. Joharatnam-Hogan N, Chambers P, Dhatariya K, et al. A guideline for the outpatient management of glycaemic control in people with cancer. *Diabet Med* 2021 Jul 9:e14636. doi: 10.1111/dme.14636. Epub ahead of print.
76. Gallo M, Clemente G, Cristiano Corsi D, et al. An integrated care pathway for cancer patients with diabetes: A proposal from the Italian experience. *Diabetes Res Clin Pract* 2020;159:107721. doi: 10.1016/j.diabres.2019.04.036.
77. Al-Taie A, Izzettin FV, Sancar M, et al. Impact of clinical pharmacy recommendations and patient counselling program among patients with diabetes and cancer in outpatient oncology setting. *Eur J Cancer Care (Engl)* 2020;29(5):e13261. doi: 10.1111/ecc.13261.

For peer review only

BMJ Open

Identifying ways to improve diabetes management during cancer treatments (INDICATE): protocol for a qualitative interview study with patients and clinicians

Journal:	*BMJ Open*
Manuscript ID	bmjopen-2021-060402.R1
Article Type:	Protocol
Date Submitted by the Author:	21-Jan-2022
Complete List of Authors:	Ashley, Laura; Leeds Beckett University,<br>Kassim, Saifuddin ; Leeds Teaching Hospitals NHS Trust, Leeds Centre for Diabetes and Endocrinology<br>Kellar, Ian; Bradford Institute for Health Reserach, Yorkshire Quality and Safety Research Group<br>Kidd, Lisa; University of Glasgow, Nursing & Healthcare, School of Medicine, Dentistry and Nursing; University of Glasgow<br>Mair, Frances; University of Glasgow, General Practice and Primary Care<br>Matthews, Mike; PPI-representative<br>Price, Mollie; Leeds Beckett University<br>Swinson, Daniel; St James's University Hospital, Leeds Cancer Centre;<br>Taylor, Johanna; Mental Health and Addiction Research Group, Department of Health Sciences, University of York<br>Velikova, Galina; University of Leeds, Leeds Institute of Medical Research<br>Wadsley, Jonathan; Weston Park Hospital,
**Primary Subject Heading**:	Oncology
Secondary Subject Heading:	Diabetes and endocrinology
Keywords:	Adult oncology < ONCOLOGY, General diabetes < DIABETES & ENDOCRINOLOGY, QUALITATIVE RESEARCH

SCHOLARONE™
Manuscripts

Identifying ways to improve diabetes management during cancer treatments (INDICATE): protocol for a qualitative interview study with patients and clinicians

Laura Ashley[1], Saifuddin Kassim[2], Ian Kellar[3], Lisa Kidd[4], Frances S Mair[5], Michael Matthews[6], Mollie Price[1], Daniel Swinson[7], Johanna Taylor[8], Galina Velikova[7,9], Jonathan Wadsley[10,11]

[1]Leeds School of Social Sciences, Leeds Beckett University, Leeds, UK

[2]Leeds Centre for Diabetes and Endocrinology, Leeds Teaching Hospitals NHS Trust, Leeds, UK

[3]School of Psychology, University of Leeds, Leeds, UK

[4]School of Medicine, Dentistry & Nursing, University of Glasgow, Glasgow, UK

[5]Institute of Health and Wellbeing, University of Glasgow, Glasgow, UK

[6]Patient and Public Involvement (PPI) representative, Harrogate, UK

[7]Leeds Cancer Centre, Leeds Teaching Hospitals NHS Trust, Leeds, UK

[8]Department of Health Sciences, University of York, York, UK

[9]Leeds Institute of Medical Research at St. James's, School of Medicine, University of Leeds, Leeds, UK

[10]Weston Park Cancer Centre, Sheffield Teaching Hospitals NHS Foundation Trust, Sheffield, UK

[11]Deaprtment of Oncology and Metabolism, University of Sheffield, Sheffield, UK

Correspondence to

Dr Laura Ashley

Rm CL821, Calverley Building, City Campus, Leeds Beckett University, Leeds, LS1 3HE, UK

L.J.Ashley@leedsbeckett.ac.uk

Twitter: @Dr_Laura_Ashley

Word count: 4,000

For peer review only

ABSTRACT

Introduction

A large and growing number of cancer patients have comorbid diabetes. Cancer and its treatment can adversely impact glycemic management and control, and there is accumulating evidence that suboptimal glycemic control during cancer treatment is a contributory driver of worse cancer-related outcomes in patients with comorbid diabetes. Little research has sought to understand, from the perspective of patients and clinicians, how and why different aspects of cancer care and diabetes care can complicate or facilitate each other, which is key to informing interventions to improve diabetes management during cancer treatments. This study aims to identify and elucidate barriers and enablers to effective diabetes management and control during cancer treatments, and potential intervention targets and strategies to address and harness these respectively.

Methods and analysis

Qualitative interviews will be conducted with people with diabetes and comorbid cancer (n=30-40) and a range of clinicians (n=30-40) involved in caring for this patient group (e.g., oncologists, diabetologists, specialist nurses, general practitioners). Semi-structured interviews will examine participants' experiences of and perspectives on diabetes management and control during cancer treatments. Data will be analysed using Framework analysis. Data collection and analysis will be informed by the Theoretical Domains Framework, and related Theory and Techniques Tool and Behaviour Change Wheel, to facilitate examination of a comprehensive range of barriers and enablers and support identification of pertinent and feasible intervention approaches. Study dates: January 2021 – January 2023.

Ethics and dissemination

The study has approval from NHS West Midlands – Edgbaston Research Ethics Committee. Findings will be presented to lay, clinical, academic and NHS and charity service-provider audiences via dissemination of written summaries and presentations, and published in peer-reviewed journals. Findings will be used to inform development and implementation of clinical, health services and patient-management intervention strategies to optimise diabetes management and control during cancer treatments.

For peer review only

Strengths and limitations of this study

➢ Largest, and first UK-based, qualitative study to date of patients' and clinicians' views on barriers and enablers to effective diabetes management during cancer treatments.

➢ Most in-depth qualitative examination of this topic to date, with a focus on both barriers and enablers, and ways to address and harness these respectively, for both patient and clinician diabetes management during cancer treatments.

➢ Will extend previous research by interviewing a wider range of clinicians (crucially including diabetologists) and considering other cancer treatments in addition to chemotherapy.

➢ Data collection and analysis will be informed by the Theoretical Domains Framework, and related Theory and Techniques Tool and Behaviour Change Wheel, to facilitate examination of a comprehensive range of barriers and enablers and support identification of feasible intervention approaches.

➢ Recruitment is limited, in the main, to just two sites, both Yorkshire-based.

For peer review only

INTRODUCTION

A large and growing number of cancer patients have comorbid diabetes

The incidence and prevalence of diabetes mellitus are high and increasing worldwide [1,2]. In high-income countries, it is estimated 87-91% of all people with diabetes have type 2 diabetes, 7-12% type 1, and 1-3% other rarer types of diabetes [1]. In the United Kingdom (UK), it is estimated that 4.8 million people are living with diabetes, projected to increase to 5.3 million by 2025 [3]. Type 1 and type 2 diabetes are associated with an increased risk of cancer, including, collectively, cancers of the breast, colorectum, endometrium and oesophagus [4-7]. In the UK, one in two people now develop cancer in their lifetime [8]. Figures on the prevalence of pre-existing diabetes in newly diagnosed cancer patients vary (e.g., by country, cancer type) but studies typically report rates ranging from 10-20% [e.g.,9-12].

Cancer and its treatment can adversely impact glycemic management and control

For people with diabetes, maintaining good glycemic control can be a significant challenge [13,14]. This challenge may be exacerbated by cancer and its treatment, which has high potential to complicate diabetes management and glycemic control. The psychosocial sequelae of a cancer diagnosis (e.g., distress, anxiety, depression [15-17]) and the side-effects of some cancer treatments (e.g., vomiting, fatigue, pain, [18,19]) could impede diabetes management behaviours such as healthy eating and blood glucose monitoring. Furthermore, some cancer and supportive treatments can directly impact blood sugar levels increasing the risk of hypo- and hyperglycemic episodes (e.g., somatostatin analogues, high-dose steroids). Though results are mixed, studies collectively indicate a deleterious effect of cancer and its treatment on diabetes management and control, which for many people is suboptimal even before diagnosis of cancer [13,14]. Research shows that during cancer treatment many people with diabetes have reduced adherence to diabetes medications and self-care behaviours that contribute to glycemic control (e.g., blood glucose monitoring, eating healthily, exercising), and have poorer glycemic control (e.g., increased HbA1c levels, diabetes treatment escalations), than pre-cancer diagnosis [20,21]. Studies have also noted that, following a cancer diagnosis, some people with diabetes undergo less diabetes-related screening aimed at mitigation of diabetic complications (e.g., retinal screening, low-density lipoprotein tests) [22-24].

Suboptimal diabetes management and control during cancer treatment is associated with worse outcomes

Some studies have found cancer patients with pre-existing diabetes are more likely to experience toxicities and complications (e.g., infections) during cancer treatment [25-31], which can result in costly hospitalisations and compromise treatment completion. Moreover, numerous studies show that, compared to other cancer patients, those with pre-existing diabetes have higher perioperative and longer-term mortality [6,25-27,30-34]. Though findings are not uniform, there is accumulating evidence that suboptimal glycemic control during cancer treatment is a contributory driver of worse cancer-related outcomes in patients with comorbid diabetes [28,35-41]. Retrospective studies of cancer patients with comorbid diabetes have shown, for example, that good perioperative glycemic control is associated with reduced risk of morbidity and death following colectomy for colon cancer [35] and good glycemic control prior to neoadjuvant chemotherapy for cervical cancer is associated with superior tumour response and survival [37]. A small 12-week prospective study of cancer

For peer review only

patients with pre-existing diabetes found suboptimal glycemic control at chemotherapy outset predicted increased risk of developing an infection, hospitalisation, and chemotherapy reduction or discontinuation [41].

Additionally, suboptimal glycemic control can cause unpleasant symptoms (e.g., fatigue, inability to concentrate, increased thirst) which may reduce health-related quality of life (HRQoL). Indeed, a recent study found cancer patients with diabetes who had suboptimal glycemic control reported poorer HRQoL during chemotherapy than both cancer patients without diabetes and those with diabetes who had good glycemic control [42]. Also, declines in glycemic control and diabetes-related screening during the period of a cancer diagnosis and treatment could increase the risk of diabetic complications such as retinopathy and cardiovascular events. A study in Canada found that, compared to diabetes patients without cancer, diabetes patients with cancer had significantly more hospital visits for diabetic emergencies, skin and soft tissue infections, and cardiovascular events in the year following their cancer diagnosis [43].

Few studies have examined patients' and clinicians' views on barriers and enablers to effective diabetes management during cancer treatments

Little research has sought to identify and understand the barriers and enablers to effective diabetes management and control during cancer treatments. Understanding how and why different aspects of cancer care and diabetes care can complicate or facilitate each other, from the perspective of patients and clinicians, is key to informing clinical, health services and patient-management interventions to improve diabetes management during cancer treatments. In a survey of people with diabetes (n=37), Hershey et al. [44,45] found that patient-reported reductions in diabetes self-management activities during cancer chemotherapy were associated with greater symptom burden and lower diabetes self-efficacy. Hershey et al.'s survey also included two open-ended questions about the impact of cancer on diabetes, which revealed that many patients prioritised cancer care over diabetes care, with some reporting advice from primary-care providers not to be concerned with diabetes during chemotherapy. In a focus-group with patients (n=5), Hershey et al. [46] similarly found that patients reported that cancer treatment took priority.

Hershey and colleagues also conducted focus-groups with oncology clinicians (n=20) [46], finding that oncologists generally saw diabetes management to be outside their remit, and the responsibility of primary-care, but noted poor communication between oncology and primary-care. These findings were recently corroborated by Cho et al. [47], who interviewed oncologists (n=10) and primary-care doctors and nurses (n=10) about diabetes management during cancer treatment. Cho et al. found both oncology and primary-care providers thought primary-care should be responsible for diabetes care, though noted barriers to this including very infrequent and limited communication between oncology and primary-care, and the fact that many patients reduce contact with primary-care following a cancer diagnosis.

Though these qualitative studies provide important insights into the challenges of managing diabetes during cancer treatments, they have involved a relatively small number of patients and clinicians, and are limited in scope and depth. Hershey's studies with patients [44-46] both focused on only chemotherapy cancer treatment, and predominately surveyed participants during this treatment, meaning they could not obtain perspectives on other elements of cancer treatment (e.g., radiotherapy, long-term tamoxifen) or with the benefit of reflection (i.e., looking back on completed treatment). Furthermore, with just two open-ended questions, Hershey et al.'s survey study did not

For peer review only

undertake an in-depth examination of participants' experiences. The studies with clinicians by Hershey [46] and Cho [47] both focused exclusively on type 2 diabetes and do not include the perspectives of several professions relevant to diabetes management during cancer, including diabetes doctors and specialist nurses, anaesthetists, and dieticians. Moreover, these prior studies have focused on barriers to diabetes management during cancer treatments, with limited or no focus on patients' and clinicians' perspectives on enabling factors and potential interventions. Also, current studies are exclusively USA-based, and findings may to some degree be context-specific, given differences in the organisation and financing of heath care systems globally, even within higher-income countries. We aim to extend, and address some of the limitations of, this previous qualitative work.

Our study aims to extend previous qualitative work in this area and help inform intervention development

The current qualitative interview study aims to identify and elucidate challenges and enablers to diabetes management and control during treatment for cancer, based on the experiences and perspectives of people with diabetes and comorbid cancer and health care professionals involved in their care. To facilitate examination of a comprehensive range of individual and service level barriers and enablers, and to support the identification of potential pertinent intervention approaches to address and harness these respectively, we will use the Theoretical Domains Framework (TDF) [48,49], and related Theory and Techniques Tool (TTT) [50-53] and Behaviour Change Wheel (BCW) [54,55], to inform data collection and analysis, as detailed in the Methods section.

Research questions

1. What are the patient-perceived challenges and enablers to effective self-management and control of diabetes during cancer treatments?

2. What are the clinician-perceived challenges and enablers to effective clinical management and control of diabetes during cancer treatments?

3. What are patients' suggestions for ways to support and improve self-management and control of diabetes during cancer treatments?

4. What are clinicians' suggestions for ways to support and improve clinical management and control of diabetes during cancer treatments?

5. What are potentially promising intervention targets and strategies for consideration in future research to optimise patient and/or clinician management and control of diabetes during cancer treatments?

For peer review only

METHODS AND ANALYSIS

Participants

Eligibility criteria are detailed in Table 1. We will include patients with type 1 or type 2 diabetes, and clinician interviews will enquire about differences between these patient groups. This will enable us to examine in the analysis similarities and differences in patient and clinician reported challenges and enablers to diabetes management during cancer treatments on the basis of diabetes type, and thus help inform to what extent different future interventions in this area could address both diabetes types or may need to target one or both of the types separately. We will restrict recruitment to comorbid breast, prostate, or colorectal cancer; in the UK, these are three of the four most common cancers and the largest survivor groups [56].

Table 1 Participant eligibility criteria

	Inclusion	*Exclusion*
Patients	➢ Medically-diagnosed type 1 or type 2 diabetes<br>➢ Subsequent diagnosis of breast, prostate, or colorectal cancer<br>➢ Received any type of localised or systemic National Health Service anti-cancer treatment (currently or within the last 3-years)	➢Under 18-years of age<br>➢ Clinician-estimated life expectancy of less than 3-months<br>➢ Lack capacity to provide informed consent
Clinicians	➢ Involved in providing care to above comorbid patient group (i.e., cancer patients with pre-existing diabetes) in relation to their diabetes and/or cancer	

Recruitment sites and procedures

Patients

Hospital-based recruitment

Patients will be primarily recruited from Cancer Centres in two Yorkshire-based National Health Service (NHS) Hospital Trusts. Clinical teams, possibly with Clinical Research Network support, will identify eligible patients and first approach them about the study, providing a patient information sheet. Interested patients will contact our research team directly, or, if a patient requests it, the clinical team will pass onto us patient-provided contact information and we will initiate correspondence.

For peer review only

Community-advertisement recruitment

We will also recruit patients via an advertisement flyer calling for people aged 18+ "with diabetes (type 1 or type 2), who are being treated for breast, bowel or prostate cancer, or have been in the past 3 years" to "tell us about your experiences of managing diabetes during cancer treatments". The flyer will be disseminated via social media (e.g., Twitter and Facebook accounts of the research team members), and by relevant willing UK-based charities and organisations (e.g., in newsletters). Patients who see the flyer and are interested will contact our research team directly.

Clinicians

Hospital-based recruitment

Clinicians will be recruited from the Cancer Centres, as well as other relevant hospital departments and specialties (e.g., endocrinology, anaesthesia, pharmacy, dietetics) within the participating hospitals. Our research team includes oncology and diabetes clinicians working at the participating hospital Trusts. Potentially eligible clinicians will be identified and emailed a staff information sheet by a member of the research team, or by a gatekeeper colleague who has agreed to disseminate study information. Eligible and interested clinicians contact the research team directly.

Primary-care recruitment

We will also recruit General Practitioners (GPs) and practice nurses working at general practices in Yorkshire. GPs and practice nurses will be informed about the study by local primary-care R&D teams who are willing to disseminate study information (e.g., in a CCG-newsletter), or will be emailed a staff information sheet by a member of the research team (e.g., GPs with a part-time academic post known to the research team). Eligible and interested clinicians contact the research team directly.

Sample size and sampling strategy

We will recruit and interview 30-40 patients and 30-40 clinicians. Based on our experience, this sample size will enable adequate sample diversity on key participant characteristics and allow us to reach sufficient data saturation.

We aim to recruit comparable numbers of people with breast, prostate, and colorectal cancer (n≈10-13 each cancer type), with both diabetes types represented in each cancer subgroup (2-3 patients in each cancer subgroup with type 1 diabetes, which accounts for 7-12% of all diabetes cases in high-income countries [1]). We also aim for some diversity in the sample as a whole with regard to types of cancer treatment, sociodemographic characteristics (gender, age, ethnicity), and the extent of experienced difficulties with diabetes management during cancer treatments.

We aim to recruit clinicians from a wide range of relevant professions and specialities (e.g., oncologists, radiographers, surgeons, diabetologists, specialist nurses, dieticians, GPs) and for some diversity in the sample as a whole with regard to professional seniority.

The composition of the sample will be monitored during recruitment and, if necessary and possible, targeted recruitment of under-represented groups will be undertaken.

For peer review only

Theoretical framework informing data collection and analysis

We will use the TDF [48,49] and related resources to inform data collection and analysis. The TDF synthesises key constructs in numerous theories of behaviour and behaviour change, and thus "provides a theoretical lens through which to view the cognitive, affective, social and environmental influences on behaviour" [p.2,57]. The TDF version-2 comprises 84 constructs theorised to influence behaviour (e.g., professional identity; self-efficacy; cognitive overload/tiredness) organised by 14 domains (e.g., social/professional role and identity; beliefs about consequences; environmental context and resources). These domains can be considered Mechanisms of Action (MoA), that is processes which influence behaviour, and are thus potential intervention targets.

The TDF is part of an evolving set of resources being developed by Michie et al. to promote design of more effective theory-based behavioural interventions. These resources include the TTT [50-53], which provides guidance on linking MoAs to pertinent Behaviour Change Techniques (BCTs) (e.g., verbal persuasion about capability; conserving mental resources; information about health consequences), which are the potentially 'active ingredients' in an intervention that changes behaviour. The TTT synthesises the evidence (or not) for links between 74 BCTs and 26 MoAs, which include the 14 TDF domains and the 12 most frequently occurring MoAs which did not overlap with these identified in a review of behaviour change theories [52]. The BCW [54,55] is a 'theory and evidence based' intervention development approach [58] that provides guidance on considering and identifying the function of interventions (e.g., education, persuasion, enablement) and policy categories that may support the delivery of these functions (e.g., guidelines, service provision, environmental planning) and how these both link to MoAs and/or BCTs.

The TDF and related resources have been used to inform the development and evaluation of health-focused behavioural interventions [e.g.,59-61], including informing the data collection and/or analysis of qualitative studies forming an early stage in the process of intervention development [62-65].

Data collection: interview content and procedures

Participants will take part in one semi-structured qualitative interview lasting around 45 minutes, though duration is likely to be variable depending on how much a participant wishes to say and their preferred interview pace. Participants can choose their interview date/time and mode (e.g., telephone, videocall, in person) provided arrangements adhere to current relevant government and workplace rules around COVID-19 social distancing.

Interviews will examine participants' experiences of and perspectives on diabetes management and control during cancer treatments. Early interview questions will enquire about key sociodemographic characteristics and relevant clinical (e.g., diabetes and cancer type and treatments) or professional (e.g., job-title, workplace) details. Interviews will seek to identify and elucidate patient- and clinician-perceived challenges and enablers to effective diabetes management and glycemic control in the context of cancer diagnosis and treatment, and ways to address and optimise these respectively.

Interview guides were developed, informed by: (1) previous research [e.g.,44-47]; (2) advice and feedback from the study PPI and steering groups; and lastly (3) the MoA covered by the TTT [50] which, as previously discussed, includes the TDF domains. We were mindful interviews do not become dominated by examining MoAs, especially given multiple behaviours are involved in managing diabetes (e.g., prescribing medications, following dietary advice, blood glucose self-monitoring) and to systematically examine each MoA in relation to each different behaviour that

For peer review only

may be discussed would make for an overly long, repetitive, and granular-level interview. Also, we did not want interviews to be restricted to examining only the MoAs, thereby potentially overlooking other influential factors. Thus, in an approach consistent with recent recommendations for using the TDF in qualitative studies [66], the interview guides contained at least one question or follow-up question likely to encourage discussion of challenges or enablers relevant to each MoA, rather than a highly-structured list of MoA-focused questions, one per MoA per different behaviours. Pilot interviews were undertaken with 3 clinician co-applicants and 3 PPI-group members, to refine the interview guides and hone interviewer technique. Box 1 shows sample questions from the patient and clinician interview guides. Interviews will be audio-recorded.

Box 1 Sample interview questions from the interview guides

Patient interviews

Selected opening questions to key topic areas and example follow-up questions

➢ In what ways, if any, did your diabetes management change during the time that you were/are having cancer treatment?
 – any changes to the foods you ate?
➢ In terms of managing your diabetes during cancer treatments, what have you found to be your biggest challenges?
 – has it been difficult to remember (e.g., to take tablets, self-monitor blood glucose) during treatment?
➢ How important do you feel it is to effectively manage your diabetes throughout cancer treatments?
 – is it more or less important to you than before cancer?
➢ How possible do you think it is to manage diabetes well whilst having cancer treatments?
 – because of the work/time/energy involved in undergoing cancer treatments?
➢ Did you receive any information about diabetes management during cancer treatments?
 – from who, when?
➢ What has the support been like from health care professionals in terms of managing your diabetes during the time that you were having cancer treatment?
 – how important did it seem to your (e.g., oncologist, GP) to manage your diabetes well during this time?
➢ Have any family or friends been involved in helping you to manage your diabetes during the time you were having cancer treatment?
 – how do they help?
➢ What do you think could be done to help people to manage their diabetes better during the time they are having cancer treatment?
 – why would that help?

Example cross-topic follow-up questions

- is that something you know about?
- is that something you know how to do?
- is that something that would be easy or difficult to do?
- what would prompt you to do that?

For peer review only

- what would be the reasons to do /not do that?
- and do you think your family and friends / doctors tend to think similarly?
- what would help you with that?

Clinician interviews

Selected opening questions to key topic areas and example follow-up questions

➢ Research suggests that managing diabetes can be difficult during cancer treatments, is this something you see/encounter in your role?

– are there differences in the difficulties between patients with type 1 and type 2 diabetes?

➢ As a (e.g., medical oncologist, GP), what do you see as your role in supporting clinical management of diabetes during cancer treatments?

– and in supporting patients' self-management of their diabetes?

➢ Can you tell me about how you identify this patient group – that you know you're dealing with a patient who has both cancer *and* diabetes

– once you know a patient has diabetes/is having cancer treatment, would it cause you to do anything differently?

➢ How important do you feel it is to effectively manage diabetes during a time that someone is also having cancer treatments?

– what do you see are the benefits of good / risks of poor diabetes management during cancer treatments?

➢ Are there any care protocols or clinical guidelines you follow when caring and making decisions for this group?

– do you find these useful?

➢ Do patients receive any information about or support with diabetes management during cancer treatments?

– do patients tend to ask for information/help?

➢ If there are difficulties controlling a patients' diabetes during cancer treatment what sorts of things would be done to address this?

– what about altering medications / bringing other professionals in / trying to improve patient self-management?

➢ Thinking of your work environment, what improvements would make it easier for you to support good diabetes management for this group?

– what about resources / recording and reporting systems / workflows and processes / culture?

Example cross-topic follow-up questions

- is that something you know how to do?
- is that something that would be easy or difficult to do?
- is that something you feel confident to do?
- is that something you always/usually/often do?
- how would you know if that had been done?
- and do you think your colleagues tend to think/do similarly?
- what would need to happen for you to do that / be able to do that?

For peer review only

Data analysis

Primary

The interview data will be analysed to identify and elucidate patient and clinician perceived challenges and enablers to self and clinical diabetes management and control during cancer treatments, and suggested ways to overcome and optimise these respectively. We will use the Framework method [67], guided by the stages and recommendations set out by Gale et al. [68]: (1) verbatim transcription; (2) familiarisation with the data; (3) coding; (4) developing a working analytical framework; (5) applying the framework; (6) charting data into a framework matrix (a summary of the data by analytic code/category per participant); and (7) interpreting the data.

Analysis will overlap with data collection. To ensure rigour, analysis will be an iterative, collaborative process led by the core research team members (LA,IK,LK,MP,JT), who have experience of framework analysis and using the TDF in qualitative research [e.g.,63,69,70], with input and feedback from other members of the research team and the PPI and steering groups at key points. We will use QSR-NVivo software to support analysis, and document substantive decisions during the analytic process.

Coding will use both deductive codes (i.e., based on the research questions, interview guides) and inductive codes (i.e., based on reading a sample of transcripts), which will be generated through iterative rounds of independent work and subsequent group discussion. In line with recent recommendations for using the TDF in qualitative studies [66], we do not intend to include the MoAs as *a priori* codes, but to subsequently consider inductively-generated findings about challenges and enablers against the MoAs (see below). This will guard against overlooking challenges and enablers discussed in the interviews not covered by the TTT and, as the TTT includes a substantial number of MoAs (n=26), guard against other key focuses of the analysis being overshadowed (e.g., interviewees suggestions for tackling challenges).

A working analytical framework will be iteratively developed through agreeing by consensus upon a final set of codes, and their initial organisation into categories, informed by input from the PPI-group and wider research team. Members of the PPI-group will each read a different sample of transcripts and provide coding-relevant feedback (e.g., feedback on sections that stood out to them, on barriers and enablers discussed in the interview). Other members of the research team and steering-group will be asked to review and feedback on a draft(s) of the working analytical framework.

The analytical framework will be applied to all interview transcripts, and subsequently the data will be charted into a framework matrix, including references to illustrative quotations. A proportion of transcripts will be double-coded and double-charted (≈10% at both stages), and compared and discussed, to ensure consistent application of the framework and data charting. We will identify and develop themes and sub-themes pertinent to the research questions, using the matrix to facilitate comparison within and between codes/categories and participants, and thus the identification of patterns and deviant cases in the data, including on the basis of diabetes and/or cancer type and/or treatments. Members of the PPI-group, wider research team and steering-group will input into data interpretation by reviewing and providing feedback on the matrix and a related working draft(s) of themes and sub-themes.

We envisage the patient and clinician data will initially be coded and charted separately, but subsequently synthesised as much as possible during interpretation and theme development. However, decisions about this aspect of the analysis will be made during analysis, after familiarisation with the data.

For peer review only - http://bmjopen.bmj.com/site/about/guidelines.xhtml

Secondary

In a second phase of analysis we will: (i) map the challenges and enablers identified in the framework analysis phase to the MoAs; (ii) prioritise MoAs for targeting in interventions; and (iii) determine pertinent intervention functions and BCTs to deliver these. We will map to the MoAs using current definitions (e.g.,[50]), and by independent work followed by group discussion to achieve a consensus. Target MoAs will be prioritised through consensus discussion, on the basis of the mapping results, the contextualised understanding of barriers and ways to address these provided by the framework analysis of the interview data, and views of the study PPI-group and wider research team and steering-group (which include multidisciplinary clinicians). For each prioritised MoA, we will determine, through consensus discussion, potential intervention functions and policy categories using the BCW [54,55] and pertinent BCTs using the TTT [50]. These processes are consistent with those undertaken in previous qualitative studies, including by members of our team, which have similarly sought to understand, and inform interventions to address, health care challenges (e.g., gestational diabetes [62]; deprescribing [63]; smoking in pregnancy [65]).

Patient and Public involvement (PPI)

Six PPI-representatives, with personal experience of diabetes, cancer or both conditions as a patient or carer, were involved in developing this research and helped shape the design and methods of the study. One of these PPI-representatives was a co-applicant on our grant application for funding and is a co-author of this protocol paper.

We have established a study PPI-group with 6 members, one of whom, co-applicant and protocol co-author MM, was involved in the previous study development stage. As well as PPI-group meetings, MM will also attend study steering-group meetings, helping ensure effective communication between these two groups. The PPI-group will collaborate, advise and feedback on all stages of the research including: design and piloting of the interview guides; data analysis including coding and interpreting the data; and dissemination outputs including lay summaries, presentations and journal papers. We will discuss with PPI-group members the research activities they wish to be involved in, their relevant prior experience (if any), and therefore what training they may require (e.g., practising data coding using transcripts from the pilot interviews), and arrange as and when appropriate. We will be guided by the UK Standards for Public Involvement [71], and PPI-representatives will be paid following UK National Institute for Health Research guidance [72] and reimbursed any expenses.

ETHICS AND DISSEMINATION

Approvals and ethical considerations

The study has approval from the NHS West Midlands – Edgbaston Research Ethics Committee (20/WM/0310), NHS Health Research Authority (IRAS-ID:276694) and the Leeds Beckett University Psychology Research Ethics Committee.

Participants will receive written study information which will include contact details of organisations providing diabetes- and cancer-related information and support (e.g., Macmillan helpline). Consent will be obtained in writing or verbally, depending on participant preference; verbal consent will be recorded immediately prior to the interview and stored on a separate audio-file to the interview. Participants will be informed of their right to choose, without needing to

For peer review only

provide a reason why, to not answer an interview question(s), to take a break or stop the interview at any time, and to request withdrawal of any/all of their data. Research and personal data will be handled and stored confidentially and securely in line with government and Leeds Beckett University data protection requirements and guidelines. We will protect participant anonymity and make unidentifiable all illustrative quotes used in reports of the findings. Participants will not be offered any incentives.

Dissemination

Findings will be presented to lay, clinical, academic and NHS and charity service-provider audiences via dissemination of written summaries and articles, infographics, and presentations. Findings will be published in a peer-reviewed journal(s) following COREQ and SRQR reporting-guidelines [73,74]. Study data may be made available on reasonable request to the corresponding author.

This UK-based study will be the first published study of its kind outside the USA, and will provide the most in-depth qualitative examination of this topic to date, with a focus on both barriers and enablers, and ways to address and harness these respectively, for both patient and clinician diabetes management during cancer treatments. Our study will extend previous work by also interviewing a wider range of clinicians, crucially including diabetologists, and considering other cancer treatments in addition to chemotherapy. Furthermore, whilst previous work in this area has not been theoretically-informed, at least explicitly, we will use the TDF, TTT, and BCW [48-55] to facilitate examination of a comprehensive range of barriers and enablers and support identification of pertinent and feasible intervention approaches.

There is increasing interest worldwide in improving glycemic control during cancer treatments, evident in the recent development of interventions for people with diabetes having treatment for cancer, such as clinical guidelines in UK [75], new integrated care pathways in Italy [76], and a clinical pharmacy intervention and counselling program in Turkey [77], though intervention research in this area is in its infancy. Findings of this study will be used to inform development and implementation of clinical, health services and patient-management intervention strategies to optimise diabetes management and control during cancer treatments.

Timeline

Data collection commenced February-2021 and is projected to close August-2022.

Contributors

LA conceived the study, led on its development and design, and is Chief Investigator and lead applicant of the grant funding this study; SK, IK, LK, FM, MM, DS, JT, GV, & JW contributed to the development of the study design and protocol and are co-applicants of the funding grant; MP is the appointed study research assistant and will undertake the interviews; the roles of team members in analysis are specified in the manuscript; LA drafted this manuscript and all co-authors reviewed, provided feedback, and approved it for submission.

For peer review only

Funding

This study is funded by the National Institute for Health Research (NIHR) Research for Patient Benefit programme, Award ID: NIHR201179. The views expressed are those of the author(s) and not necessarily those of the NIHR or the Department of Health and Social Care.

Competing interests

None

References

1. Ogurtsova K, da Rocha Fernandes JD, Huang Y, et al. IDF Diabetes Atlas: Global estimates for the prevalence of diabetes for 2015 and 2040. *Diabetes Res Clin Pract* 2017;128:40-50. doi: 10.1016/j.diabres.2017.03.024.
2. Saeedi P, Petersohn I, Salpea P, et al. Global and regional diabetes prevalence estimates for 2019 and projections for 2030 and 2045: Results from the International Diabetes Federation Diabetes Atlas, 9th edition. *Diabetes Res Clin Pract* 2019;157:107843. doi: 10.1016/j.diabres.2019.107843.
3. www.diabetes.org.uk/about_us/news/diabetes-prevalence-2019 (accessed Dec 2021)
4. Harding JL, Shaw JE, Peeters A, et al. Cancer risk among people with type 1 and type 2 diabetes: disentangling true associations, detection bias, and reverse causation. *Diabetes Care* 2015;38(2):264-70. doi: 10.2337/dc14-1996.
5. Lega IC, Lipscombe LL. Review: Diabetes, obesity, and cancer-pathophysiology and clinical implications. *Endocr Rev* 2020;41(1):bnz014. doi: 10.1210/endrev/bnz014.
6. Ling S, Brown K, Miksza JK, et al. Risk of cancer incidence and mortality associated with diabetes: A systematic review with trend analysis of 203 cohorts. *Nutr Metab Cardiovasc Dis* 2021;31(1):14-22. doi: 10.1016/j.numecd.2020.09.023.
7. Marley J, Nicholl BI, Macdonald S, et al. Associations between long-term conditions and upper gastrointestinal cancer incidence: A prospective population-based cohort of UK Biobank participants. *J Comorb* 2021;11:26335565211056136. doi: 10.1177/26335565211056136.
8. Ahmad AS, Ormiston-Smith N, Sasieni PD. Trends in the lifetime risk of developing cancer in Great Britain: comparison of risk for those born from 1930 to 1960. *Br J Cancer* 2015;112(5):943-7. doi: 10.1038/bjc.2014.606.
9. van de Poll-Franse LV, Houterman S, Janssen-Heijnen ML, et al. Less aggressive treatment and worse overall survival in cancer patients with diabetes: a large population based analysis. *Int J Cancer* 2007;120(9):1986-92. doi: 10.1002/ijc.22532.
10. Louwman WJ, Aarts MJ, Houterman S, et al. A 50% higher prevalence of life-shortening chronic conditions among cancer patients with low socioeconomic status. *Br J Cancer* 2010;103(11):1742-8. doi: 10.1038/sj.bjc.6605949.
11. Koo MM, Swann R, McPhail S, et al. The prevalence of chronic conditions in patients diagnosed with one of 29 common and rarer cancers: A cross-sectional study using primary care data. *Cancer Epidemiol* 2020;69:101845. doi: 10.1016/j.canep.2020.101845.

For peer review only - http://bmjopen.bmj.com/site/about/guidelines.xhtml

12. Fowler H, Belot A, Ellis L, et al. Comorbidity prevalence among cancer patients: a population-based cohort study of four cancers. *BMC Cancer* 2020;20(1):2. doi: 10.1186/s12885-019-6472-9.
13. Stark Casagrande S, Fradkin JE, Saydah SH, et al. The prevalence of meeting A1C, blood pressure, and LDL goals among people with diabetes, 1988-2010. *Diabetes Care* 2013;36(8):2271-9. doi: 10.2337/dc12-2258.
14. Blonde L, Aschner P, Bailey C, et al. Gaps and barriers in the control of blood glucose in people with type 2 diabetes. *Diab Vasc Dis Res* 2017;14(3):172-83. doi: 10.1177/1479164116679775.
15. Bultz BD, Carlson LE. Emotional distress: the sixth vital sign--future directions in cancer care. *Psychooncology* 2006;15(2):93-5. doi: 10.1002/pon.1022.
16. Zhu J, Fang F, Sjölander A, et al. First-onset mental disorders after cancer diagnosis and cancer-specific mortality: a nationwide cohort study. *Ann Oncol* 2017;28(8):1964-9. doi: 10.1093/annonc/mdx265.
17. Carlson LE, Zelinski EL, Toivonen KI, et al. Prevalence of psychosocial distress in cancer patients across 55 North American cancer centers. *J Psychosoc Oncol* 2019;37(1):5-21. doi: 10.1080/07347332.2018.1521490.
18. Pearce A, Haas M, Viney R, et al. Incidence and severity of self-reported chemotherapy side effects in routine care: A prospective cohort study. *PLoS One* 2017;12(10):e0184360. doi: 10.1371/journal.pone.0184360.
19. García-Chías B, Figuero E, Castelo-Fernández B, et al. Prevalence of oral side effects of chemotherapy and its relationship with periodontal risk: a cross sectional study. *Support Care Cancer* 2019;27(9):3479-90. doi: 10.1007/s00520-019-4650-6.
20. Pettit S, Cresta E, Winkley K, et al. Glycaemic control in people with type 2 diabetes mellitus during and after cancer treatment: A systematic review and meta-analysis. *PLoS One* 2017;12(5):e0176941. doi: 10.1371/journal.pone.0176941.
21. Pinheiro LC, Kaur H, Nilo D, et al. Determining the impact of a cancer diagnosis on diabetes management: a systematic literature review. *Am J Clin Oncol* 2019;42(11):870-83. doi: 10.1097/COC.0000000000000612.
22. Yao N, Camacho FT, Chukmaitov AS, et al. Diabetes management before and after cancer diagnosis: missed opportunity. *Ann Transl Med* 2015;3(5):72. doi: 10.3978/j.issn.2305-5839.2015.03.52.
23. Pinheiro LC, Soroka O, Kern LM, et al. Diabetes care management patterns before and after a cancer diagnosis: A SEER-Medicare matched cohort study. *Cancer* 2020;126(8):1727-35. doi: 10.1002/cncr.32728.
24. Pinheiro LC, Soroka O, Kern LM, et al. Racial disparities in diabetes care among incident breast, prostate, and colorectal cancer survivors: a SEER Medicare study. *J Cancer Surviv* 2021 Mar 4. doi: 10.1007/s11764-021-01003-z. Epub ahead of print.
25. Srokowski TP, Fang S, Hortobagyi GN, et al. Impact of diabetes mellitus on complications and outcomes of adjuvant chemotherapy in older patients with breast cancer. *J Clin Oncol* 2009;27(13):2170-6. doi: 10.1200/JCO.2008.17.5935.
26. Zaorsky NG, Shaikh T, Ruth K, et al. Prostate cancer patients with unmanaged diabetes or receiving insulin experience inferior outcomes and toxicities after treatment with radiation therapy. *Clin Genitourin Cancer* 2017;15(2):326-35.e3. doi: 10.1016/j.clgc.2016.08.020.

For peer review only - http://bmjopen.bmj.com/site/about/guidelines.xhtml

27. Stein KB, Snyder CF, Barone BB, et al. Colorectal cancer outcomes, recurrence, and complications in persons with and without diabetes mellitus: a systematic review and meta-analysis. *Dig Dis Sci* 2010;55(7):1839-51. doi: 10.1007/s10620-009-0944-8.
28. Iavazzo C, McComiskey M, Datta M, et al. Preoperative HBA1c and risk of postoperative complications in patients with gynaecological cancer. *Arch Gynecol Obstet* 2016;294(1):161-4. doi: 10.1007/s00404-015-3983-8.
29. Lopez-de-Andres A, Jimenez-Trujillo I, Hernandez-Barrera V, et al. Association of type 2 diabetes with in-hospital complications among women undergoing breast cancer surgical procedures. A retrospective study using the Spanish National Hospital Discharge Database, 2013-2014. *BMJ Open* 2017;7(11):e017676. doi: 10.1136/bmjopen-2017-017676.
30. Zylla D, Gilmore G, Eklund J, et al. Impact of diabetes and hyperglycemia on health care utilization, infection risk, and survival in patients with cancer receiving glucocorticoids with chemotherapy. *J Diabetes Complications* 2019;33(4):335-9. doi: 10.1016/j.jdiacomp.2018.12.012.
31. Caputo MP, Shabani S, Mhaskar R, et al. Diabetes mellitus in major head and neck cancer surgery: systematic review and meta-analysis. *Head Neck* 2020;42(10):3031-40. doi: 10.1002/hed.26349.
32. Barone BB, Yeh HC, Snyder CF, et al. Postoperative mortality in cancer patients with preexisting diabetes: systematic review and meta-analysis. *Diabetes Care* 2010;33(4):931-9. doi: 10.2337/dc09-1721.
33. Harding JL, Andes LJ, Gregg EW, et al. Trends in cancer mortality among people with vs without diabetes in the USA, 1988-2015. *Diabetologia* 2020;63(1):75-84. doi: 10.1007/s00125-019-04991-x.
34. Jing C, Wang Z, Fu X. Effect of diabetes mellitus on survival in patients with gallbladder Cancer: a systematic review and meta-analysis. *BMC Cancer* 2020;20(1):689. doi: 10.1186/s12885-020-07139-y.
35. Jackson RS, Amdur RL, White JC, et al. Hyperglycemia is associated with increased risk of morbidity and mortality after colectomy for cancer. *J Am Coll Surg* 2012;214(1):68-80. doi: 10.1016/j.jamcollsurg.2011.09.016.
36. Kang SG, Hwang EC, Jung SI, et al. Poor preoperative glycemic control is associated with dismal prognosis after radical nephroureterectomy for upper tract urothelial carcinoma: a Korean multicenter study. *Cancer Res Treat* 2016;48(4):1293-301. doi: 10.4143/crt.2016.021.
37. Li J, Ning NY, Rao QX, et al. Pretreatment glycemic control status is an independent prognostic factor for cervical cancer patients receiving neoadjuvant chemotherapy for locally advanced disease. *BMC Cancer* 2017;17(1):517. doi: 10.1186/s12885-017-3510-3.
38. Nik-Ahd F, Howard LE, Eisenberg AT, et al. Poorly controlled diabetes increases the risk of metastases and castration-resistant prostate cancer in men undergoing radical prostatectomy: Results from the SEARCH database. *Cancer* 2019;125(16):2861-7. doi: 10.1002/cncr.32141.
39. Liang SH, Shen YC, Wu JY, et al. Impact of poor preoperative glycemic control on outcomes among patients with cervical cancer undergoing a radical hysterectomy. *Oncol Res Treat* 2020;43(1-2):10-8. doi: 10.1159/000502911.
40. Zeng X, Xu C, Cheng J, et al. Poor glycemic control might compromise the efficacy of chemotherapy in non-small cell lung cancer patients with diabetes mellitus. *Cancer Med* 2020;9(3):902-11. doi: 10.1002/cam4.2750.

41. Hershey DS, Hession S. Chemotherapy and glycemic control in patients with type 2 diabetes and cancer: a comparative case analysis. *Asia Pac J Oncol Nurs* 2017;4(3):224-32. doi: 10.4103/apjon.apjon_22_17.
42. Lavdaniti M, Michalopoulou S, Owens DA, et al. The impact of comorbid diabetes type 2 on quality of life in cancer patients undergoing chemotherapy. *Endocr Metab Immune Disord Drug Targets* 2021;21(6):1017-24. doi: 10.2174/1871530320666200729151715.
43. Worndl E, Fung K, Fischer HD, et al. Preventable diabetic complications after a cancer diagnosis in patients with diabetes: a population-based cohort study. *JNCI Cancer Spectr* 2018;2(1):pky008. doi: 10.1093/jncics/pky008.
44. Hershey DS, Tipton J, Given B, et al. Perceived impact of cancer treatment on diabetes self-management. *Diabetes Educ* 2012;38(6):779-90. doi: 10.1177/0145721712458835.
45. Hershey DS, Given B, Given C, et al. Predictors of diabetes self-management in older adults receiving chemotherapy. *Cancer Nurs* 2014;37(2):97-105. doi: 10.1097/NCC.0b013e3182888b14.
46. Goebel J, Valinski S, Hershey DS. Improving coordination of care among healthcare professionals and patients with diabetes and cancer. *Clin J Oncol Nurs* 2016;20(6):645-651. doi: 10.1188/16.CJON.645-651.
47. Cho J, Nilo D, Sterling MR, et al. Eliciting primary care and oncology provider perspectives on diabetes management during active cancer treatment. *Support Care Cancer* 2021;29(11):6881-90. doi: 10.1007/s00520-021-06264-z.
48. Michie S, Johnston M, Abraham C, et al. Making psychological theory useful for implementing evidence based practice: a consensus approach. *Qual Saf Health Care* 2005;14(1):26-33. doi: 10.1136/qshc.2004.011155.
49. Cane J, O'Connor D, Michie S. Validation of the theoretical domains framework for use in behaviour change and implementation research. *Implement Sci* 2012;7:37. doi: 10.1186/1748-5908-7-37.
50. theoryandtechniquetool.humanbehaviourchange.org/ (accessed Dec 2021)
51. Carey RN, Connell LE, Johnston M, et al. Behavior change techniques and their mechanisms of action: a synthesis of links described in published intervention literature. *Ann Behav Med* 2019;53(8):693-707. doi: 10.1093/abm/kay078.
52. Connell LE, Carey RN, de Bruin M, et al. Links between behavior change techniques and mechanisms of action: an expert consensus study. *Ann Behav Med* 2019;53(8):708-20. doi: 10.1093/abm/kay082.
53. Johnston M, Carey RN, Connell Bohlen LE, et al. Development of an online tool for linking behavior change techniques and mechanisms of action based on triangulation of findings from literature synthesis and expert consensus. *Transl Behav Med* 2021;11(5):1049-65. doi: 10.1093/tbm/ibaa050.
54. Michie S, van Stralen MM, West R. The behaviour change wheel: a new method for characterising and designing behaviour change interventions. *Implement Sci* 2011;6:42. doi: 10.1186/1748-5908-6-42.
55. Michie S, Atkins L, West R. The Behaviour Change Wheel: a guide to designing interventions. London: Silverback Publishing 2014.
56. Maddams J, Utley M, Møller H. Projections of cancer prevalence in the United Kingdom, 2010-2040. *Br J Cancer* 2012;107(7):1195-202. doi: 10.1038/bjc.2012.366.

For peer review only

57. Atkins L, Francis J, Islam R, et al. A guide to using the Theoretical Domains Framework of behaviour change to investigate implementation problems. *Implement Sci* 2017;12(1):77. doi: 10.1186/s13012-017-0605-9.
58. O'Cathain A, Croot L, Sworn K, et al. Taxonomy of approaches to developing interventions to improve health: a systematic methods overview. *Pilot Feasibility Stud* 2019;5:41. doi: 10.1186/s40814-019-0425-6.
59. Craig LE, Taylor N, Grimley R, et al. Development of a theory-informed implementation intervention to improve the triage, treatment and transfer of stroke patients in emergency departments using the Theoretical Domains Framework (TDF): the T^3 Trial. *Implement Sci* 2017;12(1):88. doi: 10.1186/s13012-017-0616-6.
60. Fahim C, Acai A, McConnell MM, et al. Use of the theoretical domains framework and behaviour change wheel to develop a novel intervention to improve the quality of multidisciplinary cancer conference decision-making. *BMC Health Serv Res* 2020;20(1):578. doi: 10.1186/s12913-020-05255-w.
61. Reidy C, Foster C, Rogers A. A facilitated web-based self-management tool for people with type 1 diabetes using an insulin pump: intervention development using the behavior change wheel and theoretical domains framework. *J Med Internet Res* 2020;22(5):e13980. doi: 10.2196/13980.
62. Martis R, Brown J, McAra-Couper J, et al. Enablers and barriers for women with gestational diabetes mellitus to achieve optimal glycaemic control - a qualitative study using the theoretical domains framework. *BMC Pregnancy Childbirth* 2018;18(1):91. doi: 10.1186/s12884-018-1710-8.
63. Scott S, Twigg MJ, Clark A, et al. Development of a hospital deprescribing implementation framework: A focus group study with geriatricians and pharmacists. *Age Ageing* 2019;49(1):102-10. doi: 10.1093/ageing/afz133.
64. Maharaj AD, Evans SM, Zalcberg JR, et al. Barriers and enablers to the implementation of multidisciplinary team meetings: a qualitative study using the theoretical domains framework. *BMJ Qual Saf* 2021;30(10):792-803. doi: 10.1136/bmjqs-2020-011793.
65. Griffiths SE, Naughton F, Brown KE. Accessing specialist support to stop smoking in pregnancy: A qualitative study exploring engagement with UK-based stop smoking services. *Br J Health Psychol* 2021 Dec 1. doi: 10.1111/bjhp.12574. Epub ahead of print.
66. McGowan LJ, Powell R, French DP. How can use of the Theoretical Domains Framework be optimized in qualitative research? A rapid systematic review. *Br J Health Psychol* 2020;25(3):677-94. doi: 10.1111/bjhp.12437.
67. Ritchie J, Lewis J, McNaughton Nicholls C, et al. Qualitative research practice: a guide for social science students and researchers (2 ed.). London: Sage 2014.
68. Gale NK, Heath G, Cameron E, et al. Using the framework method for the analysis of qualitative data in multi-disciplinary health research. *BMC Med Res Methodol* 2013;13:117. doi: 10.1186/1471-2288-13-117.
69. Schnabel S, van Wijck F, Bain B, et al. Experiences of augmented arm rehabilitation including supported self-management after stroke: a qualitative investigation. *Clin Rehabil* 2021;35(2):288-301. doi: 10.1177/0269215520956388.
70. Ahern S, Arnott B, Chatterton T, et al. Understanding parents' school travel choices: a qualitative study using the Theoretical Domains Framework. *Journal of Transport and Health* 2017;4:278-93. doi: 10.1016/j.jth.2016.11.001

71. www.invo.org.uk/wp-content/uploads/2019/11/UK-standards-for-public-involvement-v6.pdf (accessed Dec 2021)
72. www.nihr.ac.uk/documents/payment-guidance-for-researchers-and-professionals/27392 (accessed Dec 2021)
73. Tong A, Sainsbury P, Craig J. Consolidated criteria for reporting qualitative research (COREQ): a 32-item checklist for interviews and focus groups. *Int J Qual Health Care* 2007;19(6):349-57. doi: 10.1093/intqhc/mzm042.
74. O'Brien BC, Harris IB, Beckman TJ, et al. Standards for reporting qualitative research: a synthesis of recommendations. *Acad Med* 2014;89(9):1245-51. doi: 10.1097/ACM.0000000000000388.
75. Joharatnam-Hogan N, Chambers P, Dhatariya K, et al. A guideline for the outpatient management of glycaemic control in people with cancer. *Diabet Med* 2021 Jul 9:e14636. doi: 10.1111/dme.14636. Epub ahead of print.
76. Gallo M, Clemente G, Cristiano Corsi D, et al. An integrated care pathway for cancer patients with diabetes: A proposal from the Italian experience. *Diabetes Res Clin Pract* 2020;159:107721. doi: 10.1016/j.diabres.2019.04.036.
77. Al-Taie A, Izzettin FV, Sancar M, et al. Impact of clinical pharmacy recommendations and patient counselling program among patients with diabetes and cancer in outpatient oncology setting. *Eur J Cancer Care (Engl)* 2020;29(5):e13261. doi: 10.1111/ecc.13261.

For peer review only

Table 1

Standards for Reporting Qualitative Research (SRQR)[a]

No.	Topic	Item	
	**Title and abstract**		
S1	Title	Concise description of the nature and topic of the study Identifying the study as qualitative or indicating the approach (e.g., ethnography, grounded theory) or data collection methods (e.g., interview, focus group) is recommended	Page 1
S2	Abstract	Summary of key elements of the study using the abstract format of the intended publication; typically includes background, purpose, methods, results, and conclusions	Page 2
	**Introduction**		
S3	Problem formulation	Description and significance of the problem/phenomenon studied; review of relevant theory and empirical work; problem statement	Pages 4-6
S4	Purpose or research question	Purpose of the study and specific objectives or questions	Page 6
	**Methods**		
S5	Qualitative approach and research paradigm	Qualitative approach (e.g., ethnography, grounded theory, case study, phenomenology, narrative research) and guiding theory if appropriate; identifying the research paradigm (e.g., postpositivist, constructivist/ interpretivist) is also recommended; rationale[b]	Page 9
S6	Researcher characteristics and reflexivity	Researchers' characteristics that may influence the research, including personal attributes, qualifications/experience, relationship with participants, assumptions, and/or presuppositions; potential or actual interaction between researchers' characteristics and the research questions, approach, methods, results, and/or transferability	
S7	Context	Setting/site and salient contextual factors; rationale[b]	Pages 7-8
S8	Sampling strategy	How and why research participants, documents, or events were selected; criteria for deciding when no further sampling was necessary (e.g., sampling saturation); rationale[b]	Page 8
S9	Ethical issues pertaining to human subjects	Documentation of approval by an appropriate ethics review board and participant consent, or explanation for lack thereof; other confidentiality and data security issues	Pages 13-14
S10	Data collection methods	Types of data collected; details of data collection procedures including (as appropriate) start and stop dates of data collection and analysis, iterative process, triangulation of sources/methods, and modification of procedures in response to evolving study findings; rationale[b]	Pages 9-10 & 14
S11	Data collection instruments and technologies	Description of instruments (e.g., interview guides, questionnaires) and devices (e.g., audio recorders) used for data collection; if/how the instrument(s) changed over the course of the study	Pages 10-11
S12	Units of study	Number and relevant characteristics of participants, documents, or events included in the study; level of participation (could be reported in results)	Pages 7 & 8
S13	Data processing	Methods for processing data prior to and during analysis, including transcription, data entry, data management and security, verification of data integrity, data coding, and anonymization/deidentification of excerpts	Pages 12-14
S14	Data analysis	Process by which inferences, themes, etc., were identified and developed, including the researchers involved in data analysis; usually references a specific paradigm or approach; rationale[b]	
S15	Techniques to enhance trustworthiness	Techniques to enhance trustworthiness and credibility of data analysis (e.g., member checking, audit trail, triangulation); rationale[b]	
	**Results/findings**		
S16	Synthesis and interpretation	Main findings (e.g., interpretations, inferences, and themes); might include development of a theory or model, or integration with prior research or theory	
S17	Links to empirical data	Evidence (e.g., quotes, field notes, text excerpts, photographs) to substantiate analytic findings	
	**Discussion**		
S18	Integration with prior work, implications, transferability, and contribution(s) to the field	Short summary of main findings; explanation of how findings and conclusions connect to, support, elaborate on, or challenge conclusions of earlier scholarship; discussion of scope of application/ generalizability; identification of unique contribution(s) to scholarship in a discipline or field	
S19	Limitations	Trustworthiness and limitations of findings	

(*Table continues*)

Table 1

(Continued)

No.	Topic	Item	
	**Other**		
S20	Conflicts of interest	Potential sources of influence or perceived influence on study conduct and conclusions; how these were managed	Page 15
S21	Funding	Sources of funding and other support; role of funders in data collection, interpretation, and reporting	Page 15

[a]The authors created the SRQR by searching the literature to identify guidelines, reporting standards, and critical appraisal criteria for qualitative research; reviewing the reference lists of retrieved sources; and contacting experts to gain feedback. The SRQR aims to improve the transparency of all aspects of qualitative research by providing clear standards for reporting qualitative research.

[b]The rationale should briefly discuss the justification for choosing that theory, approach, method, or technique rather than other options available, the assumptions and limitations implicit in those choices, and how those choices influence study conclusions and transferability. As appropriate, the rationale for several items might be discussed together.
