# Supplementary material for: Identifying ways to improve diabetes management during cancer treatments (INDICATE): protocol for a qualitative interview study with patients and clinicians
Source: BMJ Open. 2022 Feb 22;12(2):e060402. doi: 10.1136/bmjopen-2021-060402 (PMC8867345; doi:10.1136/bmjopen-2021-060402)
Supplement: Reviewer comments [file bmjopen-2021-060402.reviewer_comments.pdf]

PEER REVIEW HISTORY

BMJ Open publishes all reviews undertaken for accepted manuscripts. Reviewers are asked to complete a checklist review form (**http://bmjopen.bmj.com/site/about/resources/checklist.pdf**) and are provided with free text boxes to elaborate on their assessment. These free text comments are reproduced below.

ARTICLE DETAILS

**TITLE (PROVISIONAL)**	Identifying ways to improve diabetes management during cancer treatments (INDICATE): protocol for a qualitative interview study with patients and clinicians
**AUTHORS**	Ashley, Laura; Kassim, Saifuddin; Kellar, Ian; Kidd, Lisa; Mair, Frances; Matthews, Mike; Price, Mollie; Swinson, Daniel; Taylor, Johanna; Velikova, Galina; Wadsley, Jonathan

This article was not externally reviewed at BMJ Open. Protocols that have been independently assessed prior to submission to BMJ Open are usually fast-tracked to publication on the grounds that further substantial changes will not be possible. This independent assessment will usually be external, independent review for both a substantial grant award from a non-commercial or government funder and ethics approval.
